# Correlations between local geoclimatic variables and hatchling body size in the sea turtles *Caretta caretta* and *Chelonia mydas*

**DOI:** 10.1186/s12862-024-02290-7

**Published:** 2024-08-15

**Authors:** Omar Rafael Regalado Fernández, Parima Parsi-Pour, John A. Nyakatura, Jeanette Wyneken, Ingmar Werneburg

**Affiliations:** 1 Senckenberg Centre for Human Evolution and Palaeoenvironment an der Universität Tübingen, Sigwartstraße 10, Tübingen, 72076 Germany; 2 Fachbereich Geowissenschaften an der Universität Tübingen, Hölderlinstraße 12, Tübingen, 72074 Germany; 3 AG Vergleichende Zoologie, Institut Für Biologie, Humboldt Universität zu Berlin, Philippstraße 12 (Haus 2), Berlin, 10115 Germany; 4 Florida Atlantic University, Glades Road 777, Boca Raton, USA

**Keywords:** Green turtle, Loggerhead sea turtle, Marine turtles, Hatchlings, Carapace, Precipitation

## Abstract

It has been widely demonstrated that air and sand temperatures influence the anatomy of sea turtle hatchlings. We examined the impact of precipitation during the nesting season on the hatchling body size of loggerhead and green turtles from 37 beaches worldwide. Longitudinal data collected between 2012 and 2018 from Florida (US) and from a sample on Bõa Vista Island (Cabo Verde) carried out in 2019 showed that loggerhead body size at hatching was negatively correlated with precipitation, while precipitation was not correlated with hatchling body size in green turtles. A meta-analysis revealed that precipitation is positively correlated with hatchling mass in loggerhead turtles, while it is positively correlated with straight carapace length and width in green turtle hatchlings. The strongest influence of precipitation was found in the middle of the incubation period of loggerhead turtles in Cabo Verde, and we posit that this is due to an increase in the uptake of water for embryonic growth. These findings highlight the great importance of understanding the correlated effects of regional environmental variables, such as precipitation, on the development of sea turtle hatchlings and will have an impact on the evaluation of ongoing conservation and climate change discussions.

**Supplementary Information:**

The online version contains supplementary material available at 10.1186/s12862-024-02290-7.

## Introduction

Sea turtles are circumtropical and subtropical vertebrates distributed nearly across the globe [1, 2]. The life history of sea turtles implies early rapid growth to minimise size-specific mortality. However, high levels of plasticity in early development are likely associated with egg size, yolk content, nest hydric environment, nest thermal environment, and underlying genetics [3]. Lower incubation temperatures prolong incubation, produce larger hatchlings with smaller residual yolks, and increase the number of males per nesting site [4, 5]. Higher incubation temperatures lead to shorter incubation times, which may minimise the probability of the clutches being attacked or drowning [6]. In the loggerhead sea turtle (*Caretta caretta),* incubation temperature is negatively correlated with hatchling mass, while sand moisture is positively correlated with hatchling mass [7]. A similar pattern has been observed in the green turtle (*Chelonia mydas*) [8].

Current climatic models estimate that as temperature rises, precipitation rates become more difficult to predict [9]. While rising temperatures may appear advantageous in terms of shorter incubation periods, more erratic precipitation may have an impact on embryonic growth and the number of hatchlings of sea turtles. Since the temperature has been shown to influence sea turtle size, it may influence their defence mechanisms [6]. Growing research shows that larger hatchlings move faster and are more likely to survive because they spend less time on the beach, a high-predation-risk location [10].

The carapace and plastron of adult sea turtles provide protection from predators in the open waters of the sea but also constrain some aspects of mobility in terrestrial and aquatic situations and impose an energetic load [11, 12]. This trade-off is particularly visible in hatchling and juvenile turtles, which experience high juvenile mortality and instead rely on hiding [13]. Sea turtle ontogeny is characterised by high juvenile mortality that decreases with increasing body size and age [14]. At sea, hatchlings are unable to dive deeply and are usually confined to surface waters where they are vulnerable to sea birds, sharks, and teleosts [13]. Turtles hide in or near algal mats that provide protection as well as food (primarily cnidarians and small crustaceans) [15]. Positive allometric growth begins shortly after hatching in both *C. caretta* and *C. mydas*, offering a defence mechanism against gape-limited predators [12].

In semiaquatic and freshwater turtles, nest soil hydric conditions have been shown to influence the size of hatchlings [16–20]. For instance, hatchlings of the red-eared slider turtle (*Trachemys scripta*) and the common snapping turtle (*Chelydra serpentina*) were found to be larger when incubated in moister substrate [16–18]. It has been suggested that moisture maintains high metabolic rates with the concomitant faster consumption of yolk by changing the osmotic potential needed for the movement of nutrients [18]. In sea turtles, it has been reported that rainfall cools the environment and decreases the incubation temperature, which in turn leads to larger hatchlings due to longer growth [19, 20].

Temperature is known to affect the embryonic growth of *C. caretta* [21, 22] and *C. mydas* [21, 23]. Body size is linked to hatchling performance, which is the pivotal factor for survival success after hatching [24]. Since concerns about the future climatic impact on sea turtles are increasing, there is an established body of literature on the influence of incubation temperature on hatchling body size in different sea turtle populations [8, 25–30].

Too much moisture can be deadly for embryos. Heavy rainfall, tropical storms, flooding, and tidal inundation can adversely affect sea turtle populations through nest destruction [31]. Some nests are lost to erosion, accretion, and tidal inundation in practically every nesting site. On two barrier islands in South Carolina, USA, between 1980 and 1982, 3–25% of the *C. caretta* nests that were deposited each year were destroyed by erosion and inundation [32]. A survey of 16 sea turtle nesting beaches in Florida between 2002 and 2012 revealed that beach erosion, inundation, and predation were the main causes of egg and hatchling mortality [33]. For example, all of the embryos in 15 of the 17 *C. caretta* nests that were laid on Sapelo Island, Georgia, between 1955 and 1957 were drowned by heavy rain [34]. In Georgia, torrential rains were also reported to have significantly caused *C. caretta* egg and hatchling deaths [35]. *C. caretta* eggs can withstand flooding (seawater and freshwater) during the middle of the incubation period, but freshly laid eggs and late eggs decrease hatching success when exposed to flooding [36]. The survival of hatchlings inside their nests is thus physically impacted by an increase in precipitation, but it also influences the size and age of the individuals.

In this study, we used data on *C. caretta* and *C. mydas* to assess the impact of both temperature and precipitation on hatchling size in populations around the world. Precipitation can also have a direct effect, as an increase in rainfall can decrease the sand temperature where nests are dug, as has been shown for *C. caretta* [19]. Our correlations show that rainfall outweighs the total impact of a rise in temperature since it cools the beach's surface, making it a more accurate predictor of body size than ambient temperature. Furthermore, precipitation interferes with the osmotic pressure required to maintain egg gas exchange [36]. Unfortunately, precipitation is more difficult to forecast locally based on temperature increases [37].

**Fig. 1:**
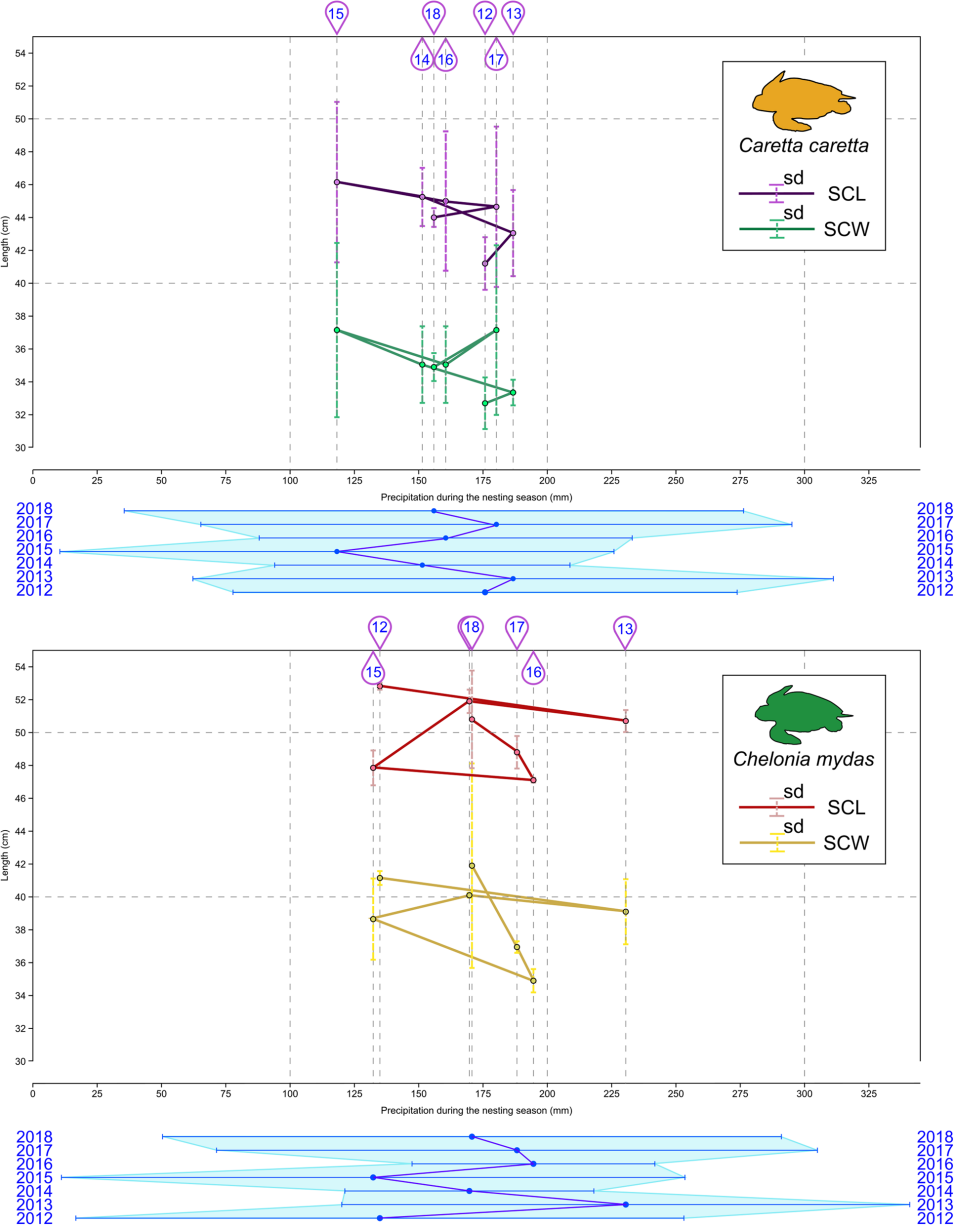
Plot illustrating the correlation between precipitation during the nesting season and the straight carapace length (SCL) and straight carapace width (SCW) in *C. caretta* and *C. mydas*. The solid line shows the time series. The longitudinal data for *Ca. caretta* and *C. mydas* include the years 2012–2018 at nesting sites on Boca Raton beach. For *C.* caretta¸, the averages and standard deviations correspond to 2012 (127 hatchlings, 13 nests), 2013 (110 hatchlings, 12 nests), 2014 (97, 11), 2015 (89,9), 2016 (124, 18), 2017 (94, 11), and 2018 (120, 12); for *C. mydas*, the averages and standard deviations correspond to 2012 (101 hatchlings, 11 nests), 2013 (130 hatchlings, 13 nests), 2014 (80, 9), 2015 (70, 7), 2016 (103, 11), 2017 (100, 10), and 2018 (66, 8). The SCL and SCW are strongly correlated. The precipitation ranges include the nesting season, which includes the earliest and latest intake dates for each nest and are illustrated below the plots. The values were taken from NOAA’s national weather service as reported for the Fort Lauderdale station

**Fig. 2:**
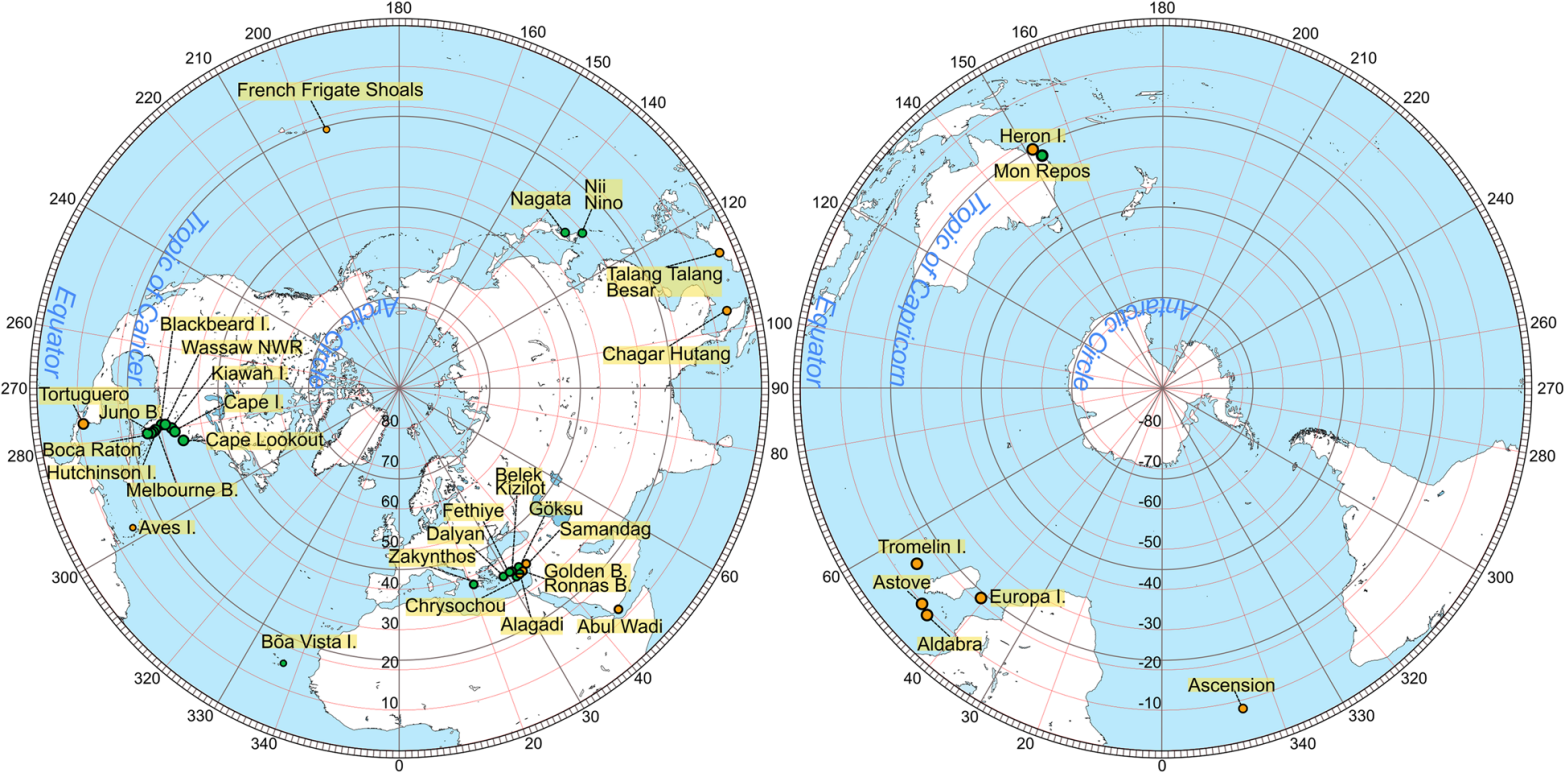
Distribution of the historical data collected in the field nesting sites of *Caretta caretta* and *Chelonia mydas* mapped on two azimuthal projections (left projection from the North Pole; right, from the South Pole). When standardising the coordinates, the *x-* and *y*-axes represent Cartesian coordinates on the azimuthal projections; the *z*-axis represents the position from the equator to the poles. Most of the historical data come from regions in the Northern Hemisphere, with an underrepresentation of the populations in the Southern Hemisphere. Historical data were used if 1) the records of the nesting site included the triplet on the hatchling size measurements (SCL, SCW and mass) or 2) the records included at least one of the hatchling body size measurements with information on the sampling size, standard deviation or range. If none of these conditions were met, the record at the nesting site was discarded

The first analysis was performed on data collected from Boca Raton, Florida, USA (Tables 1 and 2). A correlation analysis between hatchling size, measured by straight carapace length (SCL), straight carapace width (SCW) and mass (m), and geoclimatic variables was performed (Fig. 1). In a second analysis, the Florida dataset was put into a wider context by comparing the hatchling sizes against geoclimatic variables from 19 beaches for *C. caretta* [3, 31, 38–48] (Table 3, Fig. 2) and 17 beaches for *C. mydas* [2, 6, 41, 43, 49–59] (Table 4, Fig. 2). Finally, a third analysis was conducted on Cabo Verde, where the hatchlings of *C. caretta* were measured between September and October 2019 after they were laid during the dry season to determine whether the few days of precipitation had an impact on hatchling size (Table 5). We show that temperature and precipitation affect hatchling morphology but do so somewhat differently in *C. caretta* than in *C. mydas*.

**Table 1:** Climatic variables and hatchling body size of *Caretta caretta* from Florida populations

Year	Climatic variables				Hatchling body size		
	AP (mm)	AAT (C)	NSAT (C)	NSP (mm)	SCL (mm) ± s.d	SCW(mm) ± s.d	m (g) ± s.d
2012	112.4	25.1	26.8	176.6	41.2 ± 1.6	32.7 ± 1.57	18.75 ± 1.91
2013	156.8	25.2	26.2	186.7	43.05 ± 2.62	33.35 ± 0.78	18 ± 1.98
2014	124.7	25.4	27.2	156.3	45.25 ± 1.77	35.05 ± 2.33	19.85 ± 1.91
2015	117.1	25.9	27.5	112.1	46.15 ± 4.88	37.15 ± 5.3	21.05 ± 5.87
2016	143.8	25.4	27.3	160.5	45 ± 4.24	35.05 ± 2.33	19.85 ± 1.91
2017	135.6	25.5	27.1	141	44.65 ± 4.88	37.15 ± 5.16	21.3 ± 7.21
2018	123	25.3	26.7	155.9	44 ± 0.57	34.9 ± 0.85	18.7 ± 0.41

**Table 2:** Climatic variables and hatchling body size of *Chelonia mydas* from Florida populations

Year	Climatic variables				Hatchling body size		
	AP (mm)	AAT (C)	NSAT (C)	NSP (mm)	SCL (mm) ± s.d	SCW(mm) ± s.d	m (g) ± s.d
2012	112.4	25.1	26.7	134.9	52.82 ± 0.21	41.15 ± 0.42	25.55 ± 0.35
2013	156.8	25.2	27.4	230.5	50.7 ± 0.67	39.1 ± 1.98	23.35 ± 1.63
2014	124.7	25.4	28.2	169.7	51.9 ± 0.71	40.1 ± 0.14	24.3 ± 1.7
2015	117.1	25.9	28.4	132.3	47.85 ± 1.06	38.65 ± 2.47	21.55 ± 2.5
2016	143.8	25.4	28.1	194.6	47.1 ± 0.14	34.9 ± 0.71	21.55 ± 2.06
2017	135.6	25.5	28.1	188.2	48.8 ± 0.99	36.95 ± 0.35	24.45 ± 1.34
2018	123	25.3	28	170.7	50.8 ± 2.97	41.9 ± 6.22	24.8 ± 6.22

**Table 3:** Summary of the worldwide historical data on the hatchling body size of *Caretta caretta* collected at the nesting site. The summary includes data collected as part of this research. Values marked with a lozenge (◊) were reconstructed using simple linear regressions with a standard deviation estimated algebraically (see text)

Location	Country	Period	Year	Latitude	Longitude	AAT [°C] (local)	NSAT [°C] (local)	NSP [mm] (local)	SCL [mm ± sd]	SCW [mm ± sd]	m [g ± sd]
Zakynthos	GRC	Nesting season 1977, 1978, 1979 (here the average for 1978 is taken) (end of May, end of July)	1978	37.78	20.89	17.6	26.2	9.54	40.4 ± 0.7	33.9 ± 0.7	◊17.09 ± 2.16
Chrysochou Bay	CYP	Nesting season 1989	1989	35.09	32.38	17.29	22.3	8.97	40.9 ± 2.12	◊31.95 ± 2.05	16.3 ± 2.14
Goksu Delta	TUR	Nesting season (27 May to 27 September)	1992	36.17	34.04	17.9	24.7	141.9	39.1 ± 1.6	◊30.63 ± 1.34	◊16.48 ± 2.08
Fethiye	TUR	Nesting season (May to September)	1993	36.41	29.04	18	24.3	0	39.8 ± 0.19	30.1 ± 0.18	◊16.81 ± 2.26
Kizilot	TUR	ND	1996	36.71	31.56	18.4	27.3	0	39.7 ± 2.24	30.1 ± 1.5	◊16.76 ± 2.53
Belek	TUR	20 July to 18 September	1996	36.84	31.08	18.4	26.6	0.17	41.3 ± 2.02	31.44 ± 2.08	15.7 ± 1.66
Alagadi	CYP	Nesting season	1997	36.28	34.98	18.13	24.8	7.31	39.97 ± 2.67	30.37 ± 2.37	15.29 ± 3.97
Wassaw NWR and Blackbeard Island NWR	USA	2000 to 2003, mid-May until early August, eggs hatch from late June until early October	2002	31.87	-80.97	19.56	25.0	122.24	44.3 ± 0.2	◊34.42 ± 2.26	18.7 ± 0.3
Cape Lookout	USA	Early, middle and late phases of the 2002 nesting season	2002	34.6	-76.5	17.56	24.0	205.49	48.65 ± 2.18	38.88 ± 2.74	23.64 ± 3.6
Cape Island	USA	Early, middle and late phases of the 2002 nesting season	2002	33	-79.5	19.22	25.7	130.75	45.74 ± 1.11	35.05 ± 1.31	19.87 ± 1.33
Kiawah Island	USA	Early, middle and late phases of the 2002 nesting season	2002	32.5	-80	19.22	25.7	130.75	45.28 ± 1.27	34.84 ± 1.13	19.25 ± 1.29
Wassaw NWR	USA	Early, middle and late phases of the 2002 nesting season	2002	31.5	-81.2	19.56	25.0	122.24	45.29 ± 2.06	35.24 ± 1.88	19.31 ± 1.59
Melbourne Beach	USA	Early, middle and late phases of the 2002 nesting season	2002	28.87	-80.56	22.72	26.5	154.69	44.79 ± 1.51	35.16 ± 1.14	18.03 ± 1.08
Hutchinson Island	USA	Early, middle and late phases of the 2002 nesting season	2002	27.35	-80.25	25.44	27.4	124.14	44.31 ± 1.66	34.1 ± 1.5	18.77 ± 1.67
Juno Beach	USA	Early, middle and late phases of the 2002 nesting season	2002	26.87	-80.05	24.50	27.5	203.39	44.15 ± 1.31	33.48 ± 1.74	18.65 ± 1.55
Boca Raton	USA	Early, middle and late phases of the 2002 nesting season	2002	26.36	-80.06	24.89	27.4	245.30	44.4 ± 1.18	33.88 ± 1.46	18.63 ± 2.12
Dalyan	TUR	Nesting season (May to September)	2004	36.48	28.37	18.1	24.2	1.58	40.52 ± 0.84	◊31.67 ± 2.07	14.42 ± 1.24
Fethiye	TUR	Nesting season (May to September)	2004	36.41	29.04	18.1	24.2	1.58	39.46 ± 1.51	◊30.89 ± 2.03	14.12 ± 1.46
Goksu Delta	TUR	Nesting season (May to September)	2004	36.17	34.04	19.9	25.2	174.9	40.95 ± 2.12	◊31.98 ± 2.09	14.96 ± 1.48
Dalyan	TUR	June to early August (natural nest)	2004	36.48	28.37	18.1	25.8	0.25	40.4 ± 1.52	31.6 ± 1.4	14.6 ± 1.59
Dalyan	TUR	June to early August (relocated nests)	2004	36.48	28.37	18.1	25.8	0.25	40.5 ± 1.33	31.5 ± 1.09	14.5 ± 1.42
Dalyan	TUR	June to early August (natural nest) (scute deviations)	2004	36.48	28.37	18.1	25.8	0.25	40.6 ± 1.69	31.8 ± 1.36	15.1 ± 1.92
Dalyan	TUR	June to early August (relocated nests) (scute deviations)	2004	36.48	28.37	18.1	25.8	0.25	40.1 ± 1.33	31.4 ± 1.11	14.5 ± 1.41
Bõa Vista	CPV	Late June to early October	2006	16.03	22.7	23.20	24.7	92.985	41.93 ± 1.46	32.07 ± 1.18	17.23 ± 1.66
Mon Repos	AUS	5 Jan to 11 Feb	2010	-24.48	152.27	27.33	30.2	181	43.2 ± 0.3	35 ± 0.7	19.59 ± 0.7
Boca Raton	USA	19 June to 30 September	2012	26.36	-80.06	25.06	26.6	153.8	44 ± 1.61	34.21 ± 1.58	18.27 ± 1.91
Boca Raton	USA	26 June to 15 October	2013	26.36	-80.06	25.17	25.9	186.7	43.44 ± 1.72	33.73 ± 1.18	18.98 ± 1.44
Boca Raton	USA	28 June to 28 September	2014	26.36	-80.06	25.39	27.0	151.4	43.65 ± 1.38	33.84 ± 1.33	18.39 ± 1.41
Nagata	JPN	22 May to 11 June	2014	30.24	130.26	19.3	21.5	492.25	40.43 ± 1.57	32.64 ± 1.26	15.45 ± 1.85
Nii and Nino beaches on the E and W side of the Niyodo River Mouth	JPN	Early May to early August	2014	33.27	133.29	17	24.0	353.125	41.47 ± 1.43	33.37 ± 1.05	16.81 ± 1.55
Boca Raton	USA	8 July to 9 October	2015	26.36	-80.06	25.94	27.4	118.2	42.64 ± 1.9	32.35 ± 1.66	17.91 ± 2.42
Boca Raton	USA	30 June to 23 September	2016	26.36	-80.06	25.44	27.1	160.5	43.36 ± 2.58	32.94 ± 2.38	18.16 ± 1.98
Boca Raton	USA	5 July to 2 October	2017	26.36	-80.06	25.50	26.8	157.6	42.01 ± 3.44	33.22 ± 3.61	18.43 ± 3.32
Boca Raton	USA	22 June to 22 October	2018	26.36	-80.06	25.28	26.3	155.9	43.33 ± 2.5	32.98 ± 3.12	17.55 ± 2.21

**Table 4:** Summary of the worldwide historical data on the hatchling body size of *Chelonia mydas* measured at the nesting site. The summary includes data collected as part of this research. Values marked with a lozenge (◊) were reconstructed using simple linear regressions with a standard deviation estimated algebraically (see text)

Location	Country	Period	Year	Latitude	Longitude	AAT [°C] (local)	NSAT [°C] (local)	NSP [mm] (local)	SCL [mm ± sd]	SCW [mm ± sd]	m [g ± sd]
Sarawak Island	MYS	Year around, peak July to August	1951	4.76	115.01	27.38	27.38	381.30	44 ± 0.73	47.7 ± 9.72	◊21.2 ± 1.16
Ascension Island	GBR	February, March, April	1960	-7.9	-14.37	27.20	27.21	37.54	51.7 ± 2.42	41.6 ± 4.15	◊24.7 ± 3.15
Tortuguero	CRI	February, March, April	1960	10.54	-83.5	27.20	27.21	37.54	49.7 ± 1.86	42.6 ± 5.84	23.94 ± 3.09
Aldabra Atoll	SYC	Year around, peak February to October	1964	-9.42	46.34	29.90	28.48	77.00	50 ± 1.39	40.6 ± 1.59	24.06 ± 2.28
Abul Wadi beach	YEM	Year around, peak February to October	1966	12.77	44.99	32.23	32.50	5.51	46.9 ± 0.68	33.5 ± 0.44	23 ± 1.66
Astove	SYC	Year around, peak February to October	1968	-10.07	47.74	27.02	28.26	66.29	48.45 ± 0.82	39.36 ± 0.93	23.47 ± 1.85
Aldabra Atoll	SYC	Year around, peak February to October	1968	-9.42	46.34	27.02	28.26	66.29	51.31 ± 0.87	41.57 ± 0.87	29.37 ± 0.74
Aves Island	VEN	March to September, peak August	1969	-15.67	-63.62	27.80	27.00	278.95	54.6 ± 4.78	42.63 ± 8.68	27 ± 3.38
Heron Island	AUS	November to April, peal December to January	1971	-23.43	151.85	22.95	25.30	112.14	50 ± 5.14	38.32 ± 5.44	21 ± 1.05
Europa Island	FRA	Year around, peak October to February	1973	-22.36	40.37	24.20	23.36	28.60	50.9 ± 1.08	39.16 ± 6.25	25.4 ± 1.48
Tromelin Island	FRA	Year around, peak October to December	1973	-15.89	54.52	26.20	25.20	85.89	42.2 ± 0.12	31.01 ± 0.3	22.85 ± 1.61
French Frigate Shoals	FRA	May to September, peak June to July	1974	23.74	166.15	25.00	26.05	81.79	53 ± 4.97	◊41.13 ± 7.87	31 ± 4.96
Tortuguero	USA	Experiment: incubation was performed at the University of Florida on constant temperature	1980	29.64	82.35	30.00	30.00	0.00	◊39.1 ± 0.03	◊28.1 ± 0.07	19.9 ± 1.82
Alagadi	CYP	Nesting season	1997	36.28	34.98	19.20	24.98	3.00	45.68 ± 1.68	35.03 ± 1.8	19.92 ± 2.18
Heron Island	AUS	Experiment: November 1998, December 1999 (1998 has the more nests)	1998	-23.44	151.91	26.60	26.00	74.45	47.2 ± 0.4	37.6 ± 0.3	28.2 ± 0.5
Heron Island	AUS	Experiment: November 1998, December 1999 (1998 has the more nests)	1998	-23.44	151.91	26.60	30.00	74.45	47.1 ± 0.2	37.3 ± 0.2	24.2 ± 0.39
Ronas	CYP	May to August	2002	35.61	34.34	20.50	26.03	0.96	46.1 ± 1.83	35.09 ± 1.81	19.84 ± 2.02
Golden Beach	CYP	May to August	2002	35.64	34.54	20.50	26.03	0.96	46.1 ± 1.6	34.6 ± 1.73	19.97 ± 1.4
Heron Island	AUS	4 to 11 December	2007	-23.43	151.85	26.80	29.26	48.40	◊54.13 ± 6.48	32.45 ± 0.8	25.62 ± 0.76
Samandag	TUR	June to early August	2008	36.09	35.97	21.00	28.34	23.79	46 ± 1.8	34.9 ± 1.7	19.9 ± 2.1
Samandag	TUR	June to early August	2008	36.09	35.97	21.00	28.34	23.79	46 ± 1.6	35.4 ± 1.5	20.5 ± 1.5
Chagar Hutang	MYS	Experiment in situ: March to October	2009	5.81	103.01	27.10	27.33	0.00	47 ± 1.9	36.2 ± 1.6	21.5 ± 2.5
Boca Raton	USA	28 July to 11 November	2012	26.36	-80.06	25.06	26.56	153.80	50.2 ± 2.15	38.81 ± 2.7	23.57 ± 2.69
Boca Raton	USA	24 July to 22 October	2013	26.36	-80.06	25.17	25.90	186.70	50.53 ± 1.69	40.17 ± 1.3	24.21 ± 1.84
Boca Raton	USA	6 August to 14 October	2014	26.36	-80.06	25.39	27.03	151.40	51.46 ± 4.33	39.75 ± 3.99	25.35 ± 4.32
Boca Raton	USA	30 July to 2 October	2015	26.36	-80.06	25.95	27.44	118.20	48.23 ± 1.95	36.66 ± 4.31	21.89 ± 1.92
Boca Raton	USA	1 August to 14 October	2016	26.36	-80.06	25.45	27.08	160.50	50.01 ± 7.05	37.78 ± 3.07	23.94 ± 2.01
Boca Raton	USA	28 July to 12 October	2017	26.36	-80.06	25.50	26.85	157.60	49.39 ± 2.5	36.23 ± 3.6	23.72 ± 2.35
Boca Raton	USA	23 July to 22 October	2018	26.36	-80.06	25.28	26.34	155.90	50.32 ± 1.81	39.12 ± 2.39	23.38 ± 2.29

**Table 5:** The raw geoclimatic data were collected from Bõa Vista, Cabo Verde. “A” represents the temperature during the day that corresponds to mid-incubation time, “B” represents the precipitation during the day that corresponds to mid-incubation time, “C” represents the temperature during the day that corresponds to two days before mid-incubation time, and “D” represents the precipitation during the day that corresponds to two days before mid-incubation time

**Nest**	**Beach**	**Laying day**	**Emergence day**	**A (average, C)**	**B (mm)**	**C (average, C)**	**D (mm)**
**1**	Benguinho	09-07-2020	06-09-2020	23.00	0.20	24.00	0.50
**2**	Benguinho	08-07-2020	06-09-2020	23.00	0.20	24.00	0.50
**3**	Ervatão	16-07-2020	08-09-2020	24.00	0.20	25.00	0.00
**4**	Ponta Cosme	16-07-2020	06-09-2020	25.00	0.60	25.00	0.00
**5**	Ponta Cosme	16-07-2020	02-09-2020	25.00	0.00	23.00	0.20
**6**	Ervatão	16-07-2020	10-09-2020	25.00	0.20	25.00	0.60
**7**	Ponta Cosme	16-07-2020	20-09-2020	25.00	0.00	24.00	0.10
**8**	Ponta Cosme	16-07-2020	15-09-2020	24.00	0.00	25.00	0.20
**9**	Ervatão	17-07-2020	07-09-2020	24.00	0.20	25.00	0.00
**10**	Ervatão	17-07-2020	10-09-2020	25.00	0.20	25.00	0.60
**11**	Ponta Cosme	17-07-2020	13-09-2020	25.00	0.00	25.00	0.20
**12**	Ponta Cosme	18-07-2020	09-09-2020	25.00	0.20	25.00	0.60
**13**	Ponta Cosme	17-07-2020	09-09-2020	25.00	0.20	25.00	0.60
**14**	Ponta Cosme	18-07-2020	11-09-2020	25.00	0.00	24.00	0.20
**15**	Ponta Cosme	18-07-2020	16-09-2020	26.00	0.00	24.00	0.00
**16**	Ponta Cosme	18-07-2020	21-09-2020	24.00	0.00	26.00	0.00
**17**	Ervatão	19-07-2020	12-09-2020	24.00	0.00	25.00	0.20
**18**	Ervatão	18-07-2020	19-09-2020	25.00	0.00	24.00	0.10
**19**	Ervatão	19-07-2020	20-09-2020	24.00	0.00	26.00	0.00
**20**	Ponta Cosme	19-07-2020	16-09-2020	26.00	0.00	24.00	0.00
**21**	Ervatão	19-07-2020	15-09-2020	26.00	0.00	24.00	0.00
**22**	Ponta Cosme	19-07-2020	18-09-2020	25.00	0.00	24.00	0.10
**23**	Ponta Cosme	19-07-2020	13-09-2020	24.00	0.10	25.00	0.00
**24**	Ponta Cosme	20-07-2020	11-09-2020	24.00	0.00	25.00	0.20
**25**	Ervatão	20-07-2020	13-09-2020	24.00	0.10	25.00	0.00
**26**	Ponta Cosme	20-07-2020	14-09-2020	26.00	0.00	24.00	0.00
**27**	Ponta Cosme	20-07-2020	18-09-2020	24.00	0.00	26.00	0.00
**28**	Ponta Cosme	21-07-2020	10-09-2020	24.00	0.00	25.00	0.20
**29**	Ervatão	20-07-2020	12-09-2020	24.00	0.10	25.00	0.00
**30**	Benguinho	21-07-2020	15-09-2020	25.00	0.00	24.00	0.10
**31**	Ponta Cosme	21-07-2020	21-09-2020	24.00	0.20	25.00	0.00
**32**	Ervatão	21-07-2020	20-09-2020	24.00	0.00	25.00	0.00
**33**	Ervatão	21-07-2020	17-09-2020	24.00	0.00	26.00	0.00
**34**	Ponta Cosme	21-07-2020	16-09-2020	25.00	0.00	24.00	0.10
**35**	Ponta Cosme	21-07-2020	15-09-2020	25.00	0.00	24.00	0.10
**36**	Ponta Cosme	21-07-2020	09-09-2020	24.00	0.00	25.00	0.20
**37**	Ponta Cosme	22-07-2020	22-09-2020	26.00	0.00	24.00	0.00
**38**	Benguinho	07-07-2020	08-09-2020	23.00	0.20	24.00	0.50
**39**	Ponta Cosme	22-07-2020	15-09-2020	25.00	0.00	24.00	0.10
**40**	Ponta Cosme	22-07-2020	16-09-2020	24.00	0.00	26.00	0.00
**41**	Ervatão	22-07-2020	15-09-2020	25.00	0.00	24.00	0.10
**42**	Ponta Cosme	22-07-2020	19-09-2020	24.00	0.00	25.00	0.00
**43**	Ponta Cosme	22-07-2020	22-09-2020	26.00	0.00	24.00	0.00
**44**	Ervatão	22-07-2020	12-09-2020	26.00	0.00	24.00	0.00
**45**	Ervatão	23-07-2020	14-09-2020	25.00	0.00	24.00	0.10
**46**	Ervatão	23-07-2020	15-09-2020	24.00	0.00	26.00	0.00
**47**	Ervatão	23-07-2020	16-09-2020	24.00	0.00	26.00	0.00
**48**	Ponta Cosme	23-07-2020	14-09-2020	25.00	0.00	24.00	0.10
**49**	Ervatão	24-07-2020	17-09-2020	24.00	0.00	25.00	0.00
**50**	Ervatão	23-07-2020	13-09-2020	25.00	0.00	24.00	0.10
**51**	Ponta Cosme	24-07-2020	17-09-2020	24.00	0.00	25.00	0.00
**52**	Ponta Cosme	24-07-2020	16-09-2020	24.00	0.00	25.00	0.00
**53**	Ervatão	25-07-2020	18-09-2020	24.00	0.20	24.00	0.00
**54**	Ervatão	24-07-2020	19-09-2020	24.00	0.20	24.00	0.00
**55**	Ponta Cosme	25-07-2020	14-09-2020	24.00	0.00	26.00	0.00
**56**	Ponta Cosme	25-07-2020	14-09-2020	24.00	0.00	26.00	0.00
**57**	Ervatão	24-07-2020	21-09-2020	26.00	0.00	24.00	0.00
**58**	Ervatão	25-07-2020	19-09-2020	26.00	0.00	24.00	0.00
**59**	Ervatão	26-07-2020	16-09-2020	24.00	0.20	24.00	0.00
**60**	Ervatão	26-07-2020	17-09-2020	24.00	0.20	24.00	0.00
**61**	Ponta cosme	26-07-2020	19-09-2020	26.00	0.00	24.00	0.00
**62**	Ponta cosme	26-07-2020	18-09-2020	26.00	0.00	24.00	0.00
**63**	Ponta cosme	26-07-2020	21-09-2020	26.00	0.00	24.00	0.20
**64**	Ponta cosme	27-07-2020	20-09-2020	26.00	0.00	24.00	0.20
**65**	Ervatão	26-07-2020	15-09-2020	24.00	0.00	25.00	0.00
**66**	Benguinho	26-07-2020	18-09-2020	26.00	0.00	24.00	0.00
**67**	Ervatão	26-07-2020	18-09-2020	26.00	0.00	24.00	0.00
**68**	Ponta cosme	26-07-2020	20-09-2020	26.00	0.00	24.00	0.20
**69**	Ponta cosme	26-07-2020	20-09-2020	26.00	0.00	24.00	0.20
**70**	Benguinho	27-07-2020	16-09-2020	24.00	0.20	24.00	0.00
**71**	Benguinho	27-07-2020	18-09-2020	26.00	0.00	24.00	0.00
**72**	Ponta cosme	27-07-2020	20-09-2020	26.00	0.00	24.00	0.20
**73**	Ponta cosme	27-07-2020	20-09-2020	26.00	0.00	24.00	0.20
**74**	Ervatão	28-07-2020	23-09-2020	25.00	0.00	26.00	0.00
**75**	Ponta cosme	27-07-2020	24-09-2020	25.00	0.00	26.00	0.00
**76**	Ponta cosme	28-07-2020	18-09-2020	26.00	0.00	24.00	0.20
**77**	Ponta cosme	28-07-2020	17-09-2020	26.00	0.00	24.00	0.00
**78**	Benguinho	28-07-2020	21-09-2020	25.00	0.10	26.00	0.00
**79**	Ponta cosme	28-07-2020	21-09-2020	25.00	0.10	26.00	0.00
**80**	Ervatão	27-07-2020	23-09-2020	25.00	0.00	26.00	0.00
**81**	Ervatão	28-07-2020	20-09-2020	25.00	0.10	26.00	0.00
**82**	Ervatão	28-07-2020	21-09-2020	25.00	0.10	26.00	0.00
**83**	Ervatão	29-07-2020	23-09-2020	25.00	0.10	25.00	0.10
**84**	Ponta cosme	29-07-2020	23-09-2020	25.00	0.10	25.00	0.10
**85**	Ervatão	29-07-2020	21-09-2020	25.00	0.00	26.00	0.00
**86**	Ponta cosme	29-07-2020	18-09-2020	26.00	0.00	24.00	0.20
**87**	Ervatão	29-07-2020	21-09-2020	25.00	0.00	26.00	0.00
**88**	Ponta cosme	29-07-2020	20-09-2020	25.00	0.10	26.00	0.00
**89**	Ervatão	29-07-2020	23-09-2020	25.00	0.10	25.00	0.10
**90**	Ponta cosme	30-07-2020	20-09-2020	25.00	0.00	26.00	0.00
**91**	Ponta cosme	29-07-2020	19-09-2020	25.00	0.10	26.00	0.00
**92**	Ervatão	30-07-2020	20-09-2020	25.00	0.00	26.00	0.00
**93**	Ervatão	30-07-2020	23-09-2020	25.00	0.10	25.00	0.10
**94**	Ponta Cosme	30-07-2020	26-09-2020	26.00	0.00	25.00	0.10
**95**	Ponta Cosme	31-07-2020	27-09-2020	25.00	0.00	25.00	0.00
**96**	Ponta Cosme	30-07-2020	21-09-2020	25.00	0.00	26.00	0.00
**97**	Ervatão	30-07-2020	20-09-2020	25.00	0.00	26.00	0.00
**98**	Ponta Cosme	31-07-2020	25-09-2020	26.00	0.00	25.00	0.10
**99**	Ervatão	31-07-2020	23-09-2020	25.00	0.00	25.00	0.10
**100**	Ervatão	31-07-2020	22-09-2020	25.00	0.10	25.00	0.10
**101**	Ponta Cosme	31-07-2020	19-09-2020	25.00	0.00	26.00	0.00
**102**	Ervatão	31-07-2020	25-09-2020	26.00	0.00	25.00	0.10
**103**	Ponta Cosme	01-08-2020					
**104**	Ponta Cosme	01-08-2020	23-09-2020	25.00	0.00	25.00	0.00
**105**	Ervatão	01-08-2020	22-09-2020	25.00	0.00	25.00	0.00
**106**	Ervatão	01-08-2020	24-09-2020	26.00	0.00	25.00	0.10
**107**	Ponta Cosme	01-08-2020	01-10-2020	25.00	0.20	25.00	0.00
**108**	Ponta Cosme	01-08-2020	28-09-2020	25.00	0.00	26.00	0.00
**109**	Ponta Cosme	06-08-2020	27-09-2020	24.00	0.20	25.00	0.00
**110**	Ervatão	10-08-2020	07-10-2020	27.00	11.60	25.00	0.40
**111**	Ervatão	11-08-2020	02-10-2020	26.00	0.40	26.00	3.20
**112**	Ervatão	11-08-2020	06-10-2020	27.00	11.60	25.00	0.40
**113**	Ervatão	14-08-2020	09-10-2020	27.00	0.00	27.00	0.10
**114**	Ponta Cosme	14-08-2020	11-10-2020	26.00	0.00	26.00	0.00
**115**	Ponta Cosme	14-08-2020	10-10-2020	27.00	0.00	27.00	0.10
**116**	Ervatão	14-08-2020	05-10-2020	27.00	0.10	26.00	6.70
**117**	Ervatão	15-08-2020	07-10-2020	26.00	0.00	27.00	11.60
**118**	Ervatão	15-08-2020	09-10-2020	27.00	0.00	27.00	0.10
**119**	Ervatão	15-08-2020	13-10-2020	26.00	13.10	27.00	0.00
**120**	Ponta Cosme	15-08-2020	11-10-2020	26.00	0.00	26.00	0.00

## Methods

### Florida longitudinal datasets

The geoclimatic variables in this analysis included annual average air temperature (AAT), monthly average air temperature during the nesting season (NSAT), annual average cumulative precipitation (AP) and average monthly cumulative precipitation during the nesting season (NSP) for two populations of turtles, *C. caretta* (Table 1) and *C. mydas* (Table 2). We include AAT and AP because these two measurements have steadily increased in recent decades and can serve as proxies of wider climatic conditions. Still, we complement them with NSAT and NSP, given that year-round nesting rookeries are rare and that annual averages can dilute four to five months of incubation and hatchling data. Correlation analyses were performed for the morphometric data (SCL, SCW and mass) against the geoclimatic variables (AAT, NSAT, NP and NSP) (Fig. 1). The multiannual hatchling measurement data were provided by Author 4 (JW) from nesting sites in Boca Raton from 2012 to 2018. For this analysis, we used the geoclimatic data reported from the National Weather Service of the National Oceanic and Atmospheric Administration (NOAA) as collected from their Fort Lauderdale Beach station, located 31 km south of Boca Raton Beach.

### Meta-analysis of world populations

A literature review was carried out in two stages: first, to gather historical data on hatchling sizes (SCL, SCW and mass) on *C. caretta* and *C. mydas*, and second, to gather historical data on climatic variables (annual temperature, temperature, and precipitation during the nesting season). The search was performed in Scopus and Google Scholar, looking for the keywords “*Caretta*”, “*Chelonia*”, “hatchling”, and “morphometrics”, plus the places where nesting sites occur. Studies that did not precisely report where or when the hatchling measurements were taken were discarded. The first selected papers included reports of a triplet of SCL-SCW-mass, which covered a narrow geographic range. We then extended the final list to papers where at least the SCL or the SCW were reported, along with their respective range and sample sizes. This extended the geographical distribution for both datasets. Although studies on hatchling morphometrics are not common in the literature for a wide number of rookeries, data on SCL are usually reported along with other parameters, such as the size range of the individuals and the sample size. Additional data from the published literature were included when the raw data were provided by the authors upon request (Tables 3 and 4, Fig. 2).

Missing data can be dealt with when running a PCA by iterative permutation and bootstrapping on a correlation matrix. However, based on the analysis of the Florida dataset described above, there is a strong correlation between SCL and SCW and between SCL and mass; thus, a simple linear regression can model the expected SCW and mass values from SCL when data are missing. This allows estimation of the triplet SCL-SCW-mass and covers a more representative sample in terms of the geographic distribution of *C. caretta* and *C. mydas*. The reconstructed values, however, do not include a standard deviation in the analysis. Given that most papers also include information such as the range of values and the sample size, the standard deviation (SD) can be estimated algebraically as follows [60]:

$$SD\approx \frac{b-a}{2{\Phi }^{-1}\left(\frac{n-0.375}{n+0.25}\right)}$$

where *b* and *a* are the maximum and minimum values, respectively, of a sample of size *n*. The reconstructed dataset for SCL, SCW and mass allows for an estimation of the cumulative distribution function (Φ), i.e., the probability of a mean value given the normal distribution of the population mean (μ) and population SD (σ).

The reported and estimated SDs vary widely within the datasets, so to include this dispersion in the analysis, the coefficient of variance ($CV=\frac{\overline{x}}{SD }$) was calculated. This estimator may overestimate the standard deviation of large sample sizes. Nevertheless, large sample sizes are scarce when morphometric data are collected from sea turtle hatchlings, and an enhancement of this estimator requires information on the first and third quartiles (q_1_ and q_3_), which is seldom reported in the literature.

Regarding the historical climate data, few papers have reported the climatic conditions of the beaches where hatchlings were measured or collected (Tables 3 and 4). In several instances, national meteorological services provide historical data, but in others, we relied on almanacks and compilations that included the year of interest. The annual temperature (AAT), the temperature during the nesting season (NSAT) and the nesting site precipitation (NSP) data were collected from the weather stations closest to the beaches where the hatchlings were sampled. Weather data aggregators that combine raw and simulated data were used as a last resource when the information for a specific region was not available elsewhere.

### Cabo Verde study on *C. caretta* hatchlings

Data collection was carried out by Author 2 (PP) at João Barrosa beach on the south-eastern part of the island of Bõa Vista, Cabo Verde, at three nesting sites (Table 5). The data were collected from three points along the João Barrosa beach: Porto Ervatão, a bay; Ponta Cosme, a headland on the western end of the bay; and Ponta Benguinho, on the opposite side of the headland.

Daily monitoring of the beach and searching for fresh nests were followed by night shifts inside the hatcheries where the nests were relocated. The hatching process was monitored, and 20 semi-randomly selected hatchlings were measured from each of the 315 nests, where the specimens were previously collected in a bucket, which created a structural bias of capturing a preferential size (Table 6). After measuring the SCL, SCW, and mass, the turtles were immediately released to the sea. The percentage of females was estimated using a temperature-sex determination curve for the Bõa Vista *C. caretta* population. The daily average temperature and the daily cumulative precipitation were obtained from the weather data aggregator Meteoblue for the island of Bõa Vista. The data were collected during the dry season on the island. The precipitation data were retrieved from two days, one in the middle of development and the other two days before, to increase the chances of capturing a precipitation reading during the dry season. The middle of development was chosen because it has been established that the thermosensitive period, when sex is determined, occurs during the middle third of the incubation period [61]. Given that we used the data from an aggregator and we cannot separate raw from simulated data, we focused only on these two days to reduce noise. Moreover, the data was collected during a dry season, and we considered focusing on these two days instead of all the nesting seasons to reduce noise. Furthermore, PCA was performed using iterative imputation to fill in the missing data.

**Table 6:** The raw hatchling size data were collected from Bõa Vista, Cabo Verde. The percentage of females was estimated using temperature-dependent functions based on the temperature of the nest

**Nest**	**Beach**	**clutch size**	**% females**	**mass (g)**	**SCL (mm)**	**SCW (mm)**
**1**	Benguinho			17.38	44.08	
**2**	Benguinho			15.90	43.46	
**3**	Ervatão	82	91.0	16.94	43.05	32.78
**4**	Ponta Cosme	91	0.0	17.40	42.69	31.22
**5**	Ponta Cosme	81	100.0	14.30	41.10	32.88
**6**	Ervatão	96	76.5	15.75	41.42	33.30
**7**	Ponta Cosme	91	4.2	15.80	41.80	32.30
**8**	Ponta Cosme		40.3	15.31	42.45	31.98
**9**	Ervatão	114	100.0	12.13	38.50	29.73
**10**	Ervatão	83	83.7	16.23	42.48	31.92
**11**	Ponta Cosme	67	62.0	15.50	40.20	30.67
**12**	Ponta Cosme	101	98.2	15.14	40.88	30.67
**13**	Ponta Cosme	79	91.0	16.32	43.53	32.13
**14**	Ponta Cosme	86	83.7	14.61	41.86	31.76
**15**	Ponta Cosme	88	47.6	16.85	41.64	30.90
**16**	Ponta Cosme	84	11.4	10.70	37.00	30.00
**17**	Ervatão	97	83.7	15.45	41.60	30.90
**18**	Ervatão	100	25.9	14.58	41.86	32.93
**19**	Ervatão	76	25.9	14.97	41.70	32.30
**20**	Ponta Cosme	42	54.8	16.15	41.56	33.20
**21**	Ervatão	83	54.8	18.56	44.42	33.47
**22**	Ponta Cosme	78	40.3	15.87	42.79	32.50
**23**	Ponta Cosme	95	76.5	16.50	40.20	33.30
**24**	Ponta Cosme	73	98.2	17.00	43.43	33.10
**25**	Ervatão	87	76.5	15.87	41.50	31.20
**26**	Ponta Cosme	91	69.3	13.75	40.64	30.82
**27**	Ponta Cosme	91	47.6	14.28	41.14	32.03
**28**	Ponta Cosme	97	98.2	18.22	43.54	32.10
**29**	Ervatão	106	91.0	16.90	42.63	32.80
**30**	Benguinho	88	76.5	15.77	42.17	32.48
**31**	Ponta Cosme	70	33.1	12.66	39.70	31.10
**32**	Ervatão	108	40.3	18.93	44.30	
**33**	Ervatão	91	62.0	17.92	42.88	33.80
**34**	Ponta Cosme	74	69.3	16.77	42.76	31.48
**35**	Ponta Cosme	94	76.5	16.61	41.27	31.61
**36**	Ponta Cosme	89	100.0	13.56	40.37	30.31
**37**	Ponta Cosme	98	33.1	13.44	40.08	
**38**	Benguinho		25.9	17.31	41.17	32.38
**39**	Ponta Cosme	47	83.7	14.42	40.23	30.89
**40**	Ponta Cosme	109	76.5	17.82	42.22	32.84
**41**	Ervatão	102	76.5	16.59	42.47	32.91
**42**	Ponta Cosme	99	54.8	18.93	42.77	32.42
**43**	Ponta Cosme	89	33.1	15.39	41.94	31.30
**44**	Ervatão	84	40.3	17.64	43.10	33.40
**45**	Ervatão	99	98.2	15.59	42.26	31.80
**46**	Ervatão	97	91.0	15.27	39.85	29.78
**47**	Ervatão	81	83.7	18.23	42.51	33.55
**48**	Ponta Cosme	87	91.0	16.35	42.26	30.30
**49**	Ervatão	114	83.7	16.74	42.03	33.06
**50**	Ervatão	62	100.0	16.10	42.36	31.67
**51**	Ponta Cosme	90	83.7	14.94	40.90	32.10
**52**	Ponta Cosme	98	91.0	17.44	41.52	30.34
**53**	Ervatão	109	83.7	16.21	41.80	32.50
**54**	Ervatão	63	69.3	19.90	44.50	34.60
**55**	Ponta Cosme	102	100.0	17.80	43.57	34.12
**56**	Ponta Cosme	108	100.0	17.02	41.65	32.14
**57**	Ervatão	72	54.8	16.14	42.04	31.90
**58**	Ervatão	96	76.5	16.57	41.40	32.20
**59**	Ervatão	96	100.0	16.57	41.40	30.41
**60**	Ervatão	108	98.2	18.56	45.74	34.30
**61**	Ponta cosme	63	83.7	15.43	41.07	31.20
**62**	Ponta cosme	94	91.0	17.34	42.44	31.56
**63**	Ponta cosme	113	69.3	17.21	41.45	32.10
**64**	Ponta cosme	84	83.7	19.33	43.30	33.70
**65**	Ervatão	100	100.0	15.76	41.31	33.09
**66**	Benguinho	94	91.0	15.61	40.60	31.50
**67**	Ervatão	80	91.0	16.71	42.20	
**68**	Ponta cosme	98	76.5	19.08	44.70	32.80
**69**	Ponta cosme	77	76.5	15.80	43.30	31.70
**70**	Benguinho	107	98.2	18.63	43.88	32.54
**71**	Benguinho	71	98.2	16.13	41.14	31.18
**72**	Ponta cosme	98	83.7	16.13	40.60	34.00
**73**	Ponta cosme	83	83.7	16.97	41.80	31.60
**74**	Ervatão	80	69.3	15.13	41.80	31.70
**75**	Ponta cosme	68	54.8	14.70	38.20	30.10
**76**	Ponta cosme	93	100.0	19.30	43.28	33.76
**77**	Ponta cosme	89	100.0	17.20	44.08	33.51
**78**	Benguinho	91	83.7	18.80	42.47	32.67
**79**	Ponta cosme	83	83.7	19.39	43.10	32.57
**80**	Ervatão	103	33.1	15.63	42.51	32.70
**81**	Ervatão	57	91.0	19.00	45.40	33.80
**82**	Ervatão	76	83.7	16.79	41.42	32.08
**83**	Ervatão	103	76.5	16.34	42.00	33.10
**84**	Ponta cosme	94	76.5	14.50	40.64	32.18
**85**	Ervatão	77	91.0	15.74	41.31	31.40
**86**	Ponta cosme	102	100.0	17.40	41.34	31.47
**87**	Ervatão	85	91.0	16.54	42.00	33.00
**88**	Ponta cosme	119	98.2	15.75	42.30	31.70
**89**	Ervatão	90	69.3	20.83	45.32	36.00
**90**	Ponta cosme	68	76.5	16.63	42.90	33.40
**91**	Ponta cosme	101	100.0	17.80	41.60	32.10
**92**	Ervatão	79	83.7	15.97	42.00	30.90
**93**	Ervatão	83	83.7	16.10	41.90	31.40
**94**	Ponta Cosme	83	62.0	16.18	41.92	31.86
**95**	Ponta Cosme	91	54.8	16.04	41.20	32.75
**96**	Ponta Cosme	87	98.2	15.30	41.39	30.70
**97**	Ervatão	110	100.0	14.90	39.89	
**98**	Ponta Cosme	79	76.5	15.40	42.80	31.66
**99**	Ervatão	64	91.0	15.52	41.30	31.76
**100**	Ervatão	73	98.2	16.38	42.25	30.97
**101**	Ponta Cosme	90	100.0	16.71	42.12	31.46
**102**	Ervatão	86	76.5	17.96	44.50	33.65
**103**	Ponta Cosme	71	33.1			
**104**	Ponta Cosme	96	98.2	15.48	40.50	30.61
**105**	Ervatão	90	100.0	14.47	40.75	30.30
**106**	Ervatão	90	91.0	15.21	40.50	31.10
**107**	Ponta Cosme	104	40.3	13.50	39.67	32.00
**108**	Ponta Cosme	88	40.3	14.70	40.28	32.06
**109**	Ponta Cosme	96	100.0	15.29	41.68	30.74
**110**	Ervatão	77	62.0	15.81	43.69	33.07
**111**	Ervatão	86	100.0	15.17	39.80	30.36
**112**	Ervatão	98	76.5	14.45	40.37	30.20
**113**	Ervatão	83	76.5	14.88	41.55	31.65
**114**	Ponta Cosme	91	62.0	14.54	41.58	32.70
**115**	Ponta Cosme	62	69.3	12.83	39.50	30.50
**116**	Ervatão	83	100.0	17.90	43.10	33.00
**117**	Ervatão	98	98.2	18.46	43.79	34.31
**118**	Ervatão	60	83.7	15.28	40.90	31.90
**119**	Ervatão	71	54.8	14.49	40.55	30.30
**120**	Ponta Cosme	80	69.3	17.08	44.08	32.90

## Results

### Florida longitudinal datasets

The AAT remained relatively constant from 2012–2018, but the NSAT was more variable (Fig. 1). The years 2015, 2016 and 2017 were particularly dry and hot years (Fig. 1). The lowest accumulated precipitation occurred in 2015. The year 2016 was both very dry and very hot, and it is estimated that more than half of the *C. caretta* eggs died. The year 2017 was more typical in April, but it became very hot and dry during the rest of the nesting season until mid-August when typical conditions returned to the rookeries. During the period 2015–2017, the driest years, the variance in sizes was greatest for both species. The hottest nesting season for *C. caretta* occurred between 2014 and 2017, but the nesting season for *C. mydas* was, on average, one degree hotter in the same period (Fig. 2). In Florida, *C. mydas* starts nesting between the months of June and September, and *C. caretta* starts its nesting season between April and September; consequently, the weather each species experiences, as a whole, differs. The nesting seasons differed for the two species in terms of precipitation as well. Although the driest year was 2012, which was one of the most humid seasons for *C. caretta*, the NSP was drier for *C. mydas* than for *C. caretta* (Fig. 1). The year 2013 was the most humid year for both nesting seasons, and on average, the nesting season of *C. caretta* experienced less rainfall than that of *C. mydas*.

**Fig. 3:**
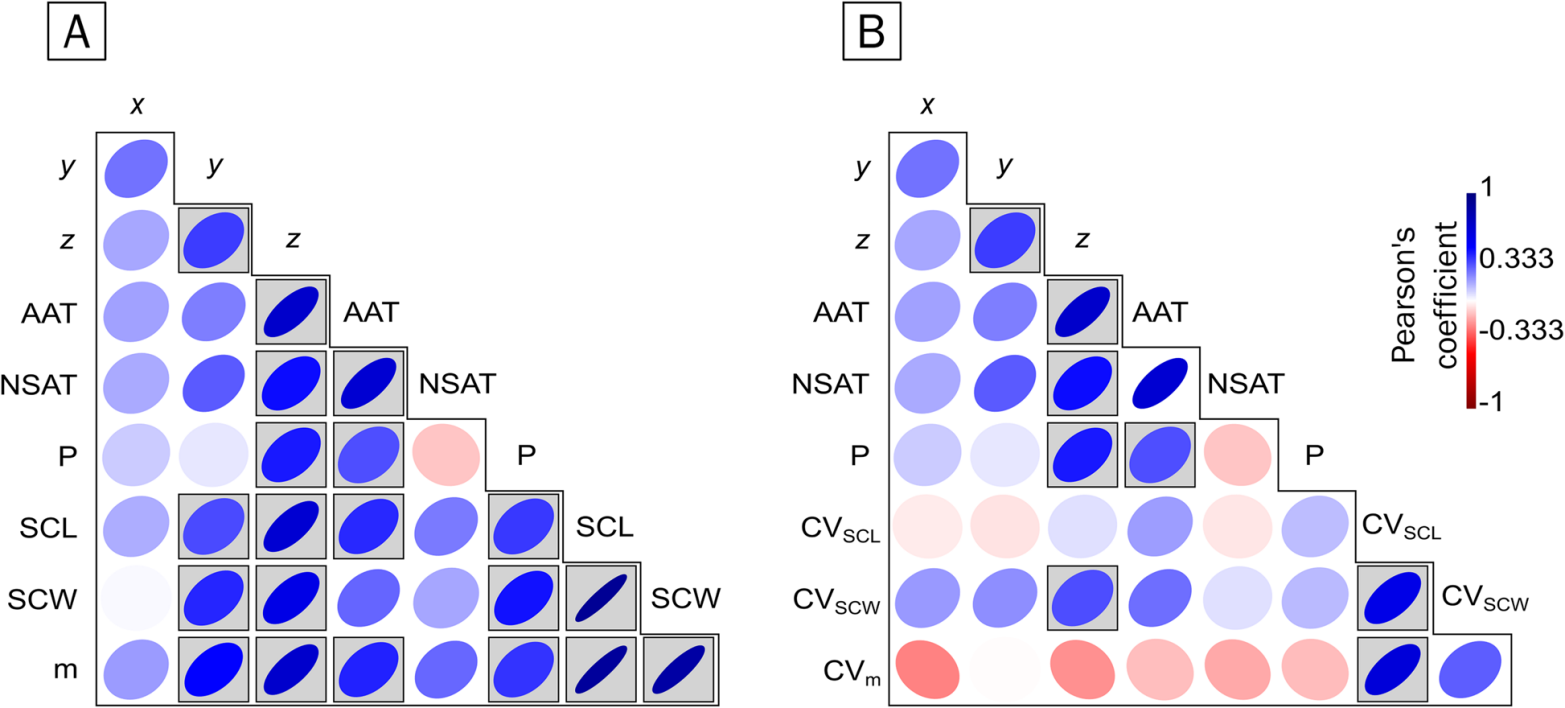
Heatmap of the correlation between geoclimatic variables and hatchling size in *Caretta caretta*. The geoclimatic variables correspond to geographic coordinates, which are decomposed into the x-axis, y-axis and z-axis, the annual air temperature (AAT), the air temperature during the nesting season (NSAT) and the precipitation during the nesting season (P). The hatchling size variables included the straight carapace length (SCL), straight carapace width (SCW) and mass. **A** The panel shows the correlation between the geoclimatic variables and the mean hatchling size. Along the z-axis, the annual air temperature and precipitation are strongly positively correlated (*p* < 0.05). **B** The panel shows the correlation between the geoclimatic variables and the coefficient of variation (CV) of the hatchling values. Only latitude and the z-axis were positively but weakly correlated with the variation in the SCW (*p* < 0.05). See *p* values in Table 7 and in the text

**Fig. 4:**
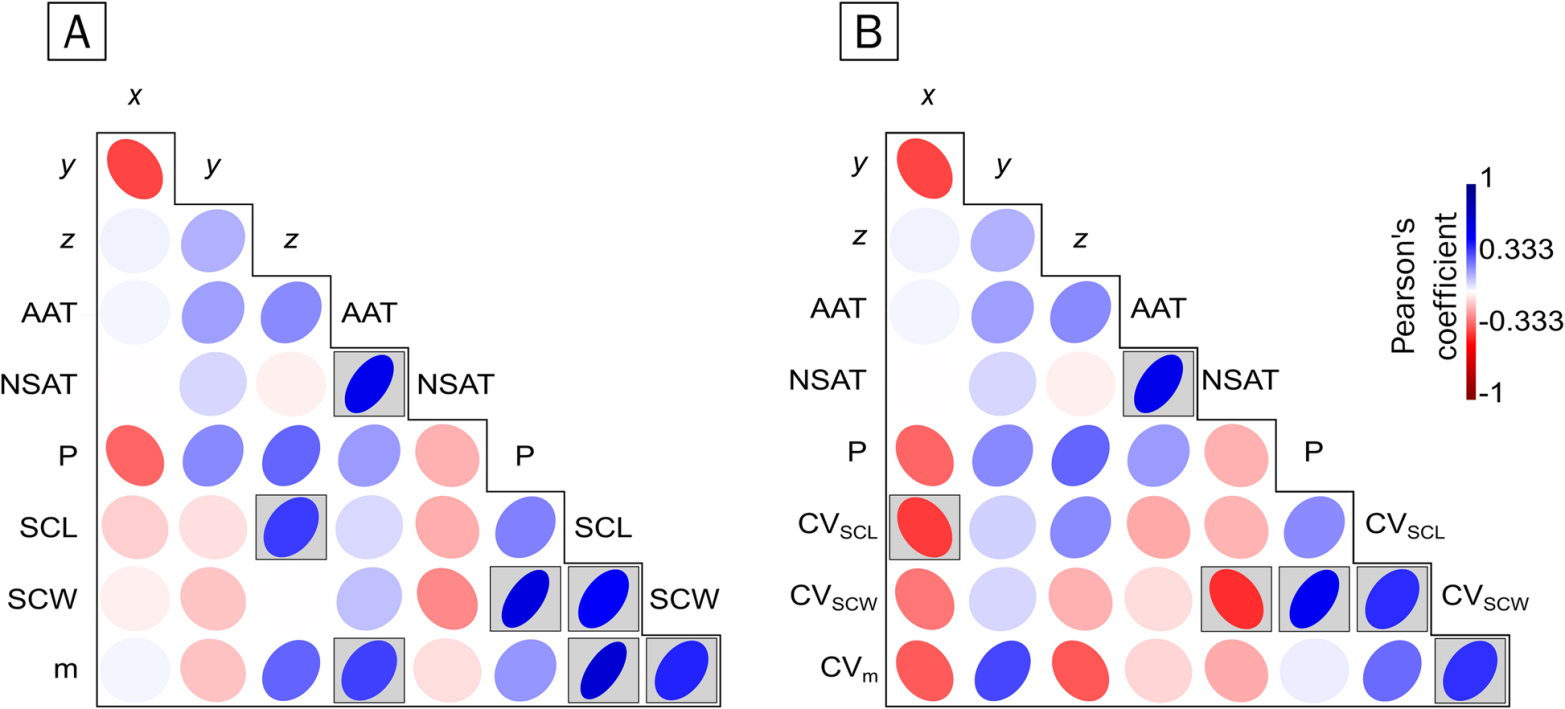
Heatmap of the correlation between geoclimatic variables and hatchling size of *Chelonia mydas*. The geoclimatic variables correspond to geographic coordinates, which are decomposed into the x-axis, y-axis and z-axis, the annual air temperature (AAT), the air temperature during the nesting season (NSAT) and the precipitation during the nesting season (P). The hatchling size variables included the straight carapace length (SCL), straight carapace width (SCW) and mass. **A** The panel shows the correlation between the geoclimatic variables and the mean hatchling size. The latitude of the z-axis is weakly positively correlated with the SCL (*p* < 0.05), the annual air temperature is correlated with the mass (*p* < 0.05), and the precipitation is correlated with the SCW (*p* < 0.05). **B** The panel shows the correlation between the geoclimatic variables and the coefficient of variation (CV) of the hatchling values. The coefficient of variation in the SCL (CV_SCL_) was negatively correlated with longitude (x-axis) (*p* < 0.05), whereas the air temperature during the nesting season (NSAT) was negatively correlated with the variation in the SCW (CV_SCW_) (*p* < 0.05), and precipitation was positively correlated with the variation in the SCW (*p* < 0.05). See *p* values in Table 8 and in the text

In the case of *C. caretta*, the precipitation during the nesting season (NSP) correlated with the mean hatchling size (SCC) (*p* = 0.0026, Table 7, Fig. 3A), SCW (*p* = 0.0055, Table 7, Fig. 3A) and mass (*p* = 0.0196, Table 7 = Fig. 3A). Still, it was not correlated with the coefficient of variation of the hatchling size metrics (Fig. 3B). The average temperature during the nesting season (NSAT) was also not correlated with any of the hatchling size metrics, i.e., averages and variation (Fig. 3). Although temperature and precipitation are correlated, at the regional level, precipitation is also a consequence of hydric, topographic, and atmospheric factors; the NSAT has a nonsignificant negative correlation (*p* = 0.5289) with NSP. The AAT is strongly positively correlated with the SCL (*p* = 0.0131, Table 7, Fig. 3A) and mass (*p* = 0.0102, Table 7, Fig. 3A). NSP was positively correlated with the average SCL (*p* = 0.0226, Table 7, Fig. 3A), SCW (*p* = 0.0055, Table 7, Fig. 3A) and mass (*p* = 0.0196, Table 7, Fig. 3A) but not with the coefficient of variation (SCL, *p* = 0.9747, SCW, *p* = 0.84656, mass, *p* = 0.2689, Supplementary material), suggesting that precipitation during the nesting season has a significant effect on the hatchling size of *C. caretta* (Fig. 3). In the case of *C. mydas*, geoclimatic variables are not as correlated with hatchling size as they are in *C. caretta*. The AAT was positively correlated with average mass (*p* = 0.04571, Table 8, 5, Fig. 4A) but not with the coefficient of variation of either of the hatchling size metrics (SCL, *p* = 0.3836; SCW, *p* = 0.73455; mass, *p* = 0.67518; Supplementary material and Fig. 4B). NSP was positively correlated with the average SCW (*p* = 0.00026, Table 8, Fig. 4A) and with the coefficient of variation of SCW (*p* = 0.00176, Supplementary material, Fig. 4B). The air temperature during the nesting season (NSAT) was negatively correlated with the coefficient of variation of the SCW (*p* = 0.02689, Supplementary material, Fig. 4B).

**Table 7:**
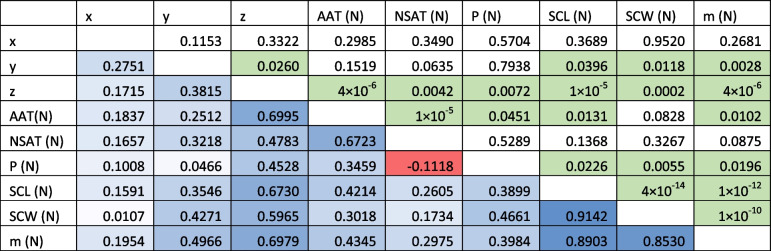
Correlation values of the meta-analysis for *C. caretta* in a matrix *p* value/correlation index. The values above the diagonal are *p* values, and the values below the diagonal are correlation indices. The geographic variables (x, y and z) correspond to the normalisation of the coordinates. The climatic variables, also normalised, are annual air temperature (AAT), nesting season air temperature (NSAT) and nesting season precipitation (P). The morphometric variables, also normalised, are straight carapace length (SCL), straight carapace width (SCW) and mass (m). The cells in the triangle below the diagonal are coloured to reflect correlation: blue indicates a positive correlation, uncoloured indicates no correlation, and red indicates a negative correlation. The cells in green indicate the values that showed statistical significance (see text)

**Table 8:**
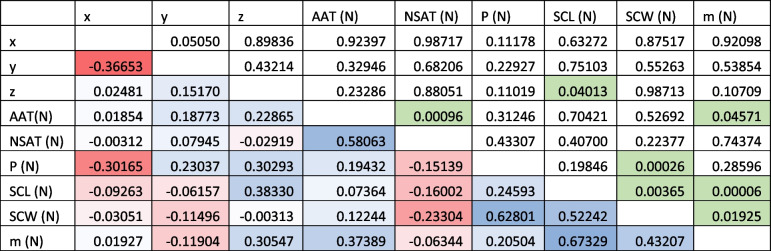
Correlation values of the meta-analysis for *C. mydas*. The geographic variables (x, y and z) correspond to the normalisation of the coordinates. The climatic variables, also normalised, are annual air temperature (AAT), nesting season air temperature (NSAT) and nesting season precipitation (P). The morphometric variables, also normalised, are straight carapace length (SCL), straight carapace width (SCW) and mass (m). The cells are coloured to indicate correlations: blue indicates a positive correlation, uncoloured indicates no correlation, and red indicates a negative correlation. The symbol “ε” represents very small values that are not zero. The cells in green indicate the values that showed statistical significance (see text)

### Meta-analysis of worldwide populations

For this analysis, we considered the effects of latitude and longitude on the populations, and the coordinates were standardised by transforming them into Cartesian pairs. The x-axis [$\text{sin}(lat)\times \text{cos}(lon)$] runs along points (0,0), the y-axis [$\text{cos}(lat)\times \text{sin}(lon)$] runs along points (0,90), and the z-axis [$\text{sin}(lat)$] runs through poles (-90,0) and (90,0). Thus, the x- and y-axes represent the positions along the surface of the Earth when viewed from an azimuthal projection, whereas the z-axis represents a location on the sphere (poles, tropics or equator). On the x-y plane, the first quadrant represents the populations sampled from the Mediterranean in the northern hemisphere, namely the populations in Greece, Cyprus, Turkey, as well as the Aden Gulf population in Abul Wadi, and the western Indian Ocean in the southern hemisphere, namely the populations in Tromelin Island, Astove, Aldabra and Europa Island off the east coast of Africa. The second quadrant represents populations from the western Pacific Ocean, with the populations of the South China and East China Seas in the northern hemisphere, and the populations from the Coral Sea in the southern hemisphere. The third quadrant represents the eastern Pacific, with only one population represented, the one in the French Frigate Shoals. The fourth quadrant represents the western Pacific coasts in South America and the northern portion of the Atlantic Ocean, which includes the Caribbean Sea and the Gulf of Mexico and the populations of Cape Verde in the northern hemisphere, and the population from the Ascension Island in the southern hemisphere (Fig. 2).

In an initial inspection of the dataset for *C. caretta*, several variables displayed a significant correlation (Table 5). The z-axis, a proxy for latitude, is positively correlated with the triplet SCL-SCW-mass. The AAT is correlated with the SCL and mass, whereas the precipitation is positively correlated with the SCL, SCW and mass. A PCA was performed on a covariance matrix in PAST 4.15 [62].

**Fig. 5:**
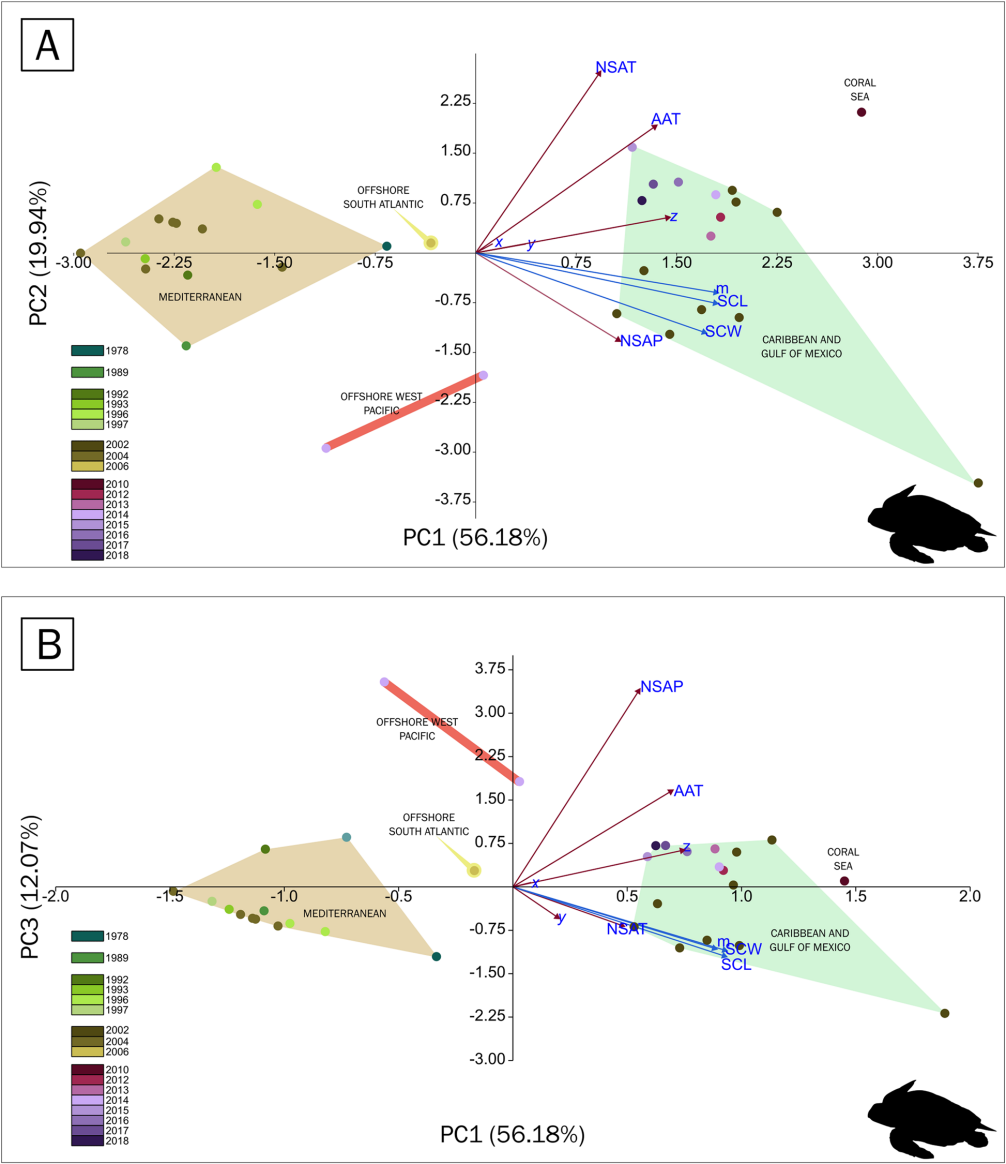
Biplots of the principal component analysis of the geoclimatic variables and hatchling sizes of *Caretta caretta*. **A** Plotting PC1 against PC2 shows that the historical records can be separated into populations based on the biogeographic marine realms: 13 samples come from the Mediterranean, 16 from the Caribbean and the Gulf of Mexico, 2 from the Offshore West Pacific, 1 from the Offshore Atlantic, and 1 from the Coral Sea. The colour scale refers to the years where the samples were collected. **B** Plotting PC1 against PC3 showing the same clusters as described in (**A**)

The first two eigenvalues explained 76% of the variance, with principal component 1 (PC1) containing mostly morphometric measurements (SCL-SCW-mass) and PC2 comprising mainly AAT and NSAT (Fig. 5A). Precipitation, SCL, SCW and mass are positively correlated. Precipitation and NSAT are likely not correlated. The latitude (z-axis) is more strongly positively correlated with the mass than with the SCL and SCW (Fig. 5).

When plotting PC1 against PC3, which has an eigenvalue of 0.84656, the same geography-based clusters were obtained (Fig. 5B). PC3 is mostly composed of NSP. The biplot shows that the precipitation at the nesting site is positively correlated with the annual temperature and the distance along the poles (z-axis). In contrast, the NSAT is closely correlated with size (SCL-SCW-mass) (Fig. 5B). The Mediterranean population was somewhat separated from the other populations, probably due to the very dry regime in the region (Fig. 5B). The initial inspection of the dataset revealed that latitude was positively correlated with SCL (*p* = 1 × 10^–5^, Table 7), SCW (*p* = 0.0002, Table 7) and mass (*p* = 4 × 10^–6^, Table 7). AAT could also be correlated to the latitude itself, as the temperature regimes change along the coasts where the populations nest. The significant correlation could be due to its not being decomposed into three vectors, as was the case with geographic coordinates. Thus, the correlation of AAT to hatchling size metrics may be an artefact of the turtle populations having different sizes along long coastlines.

**Fig. 6:**
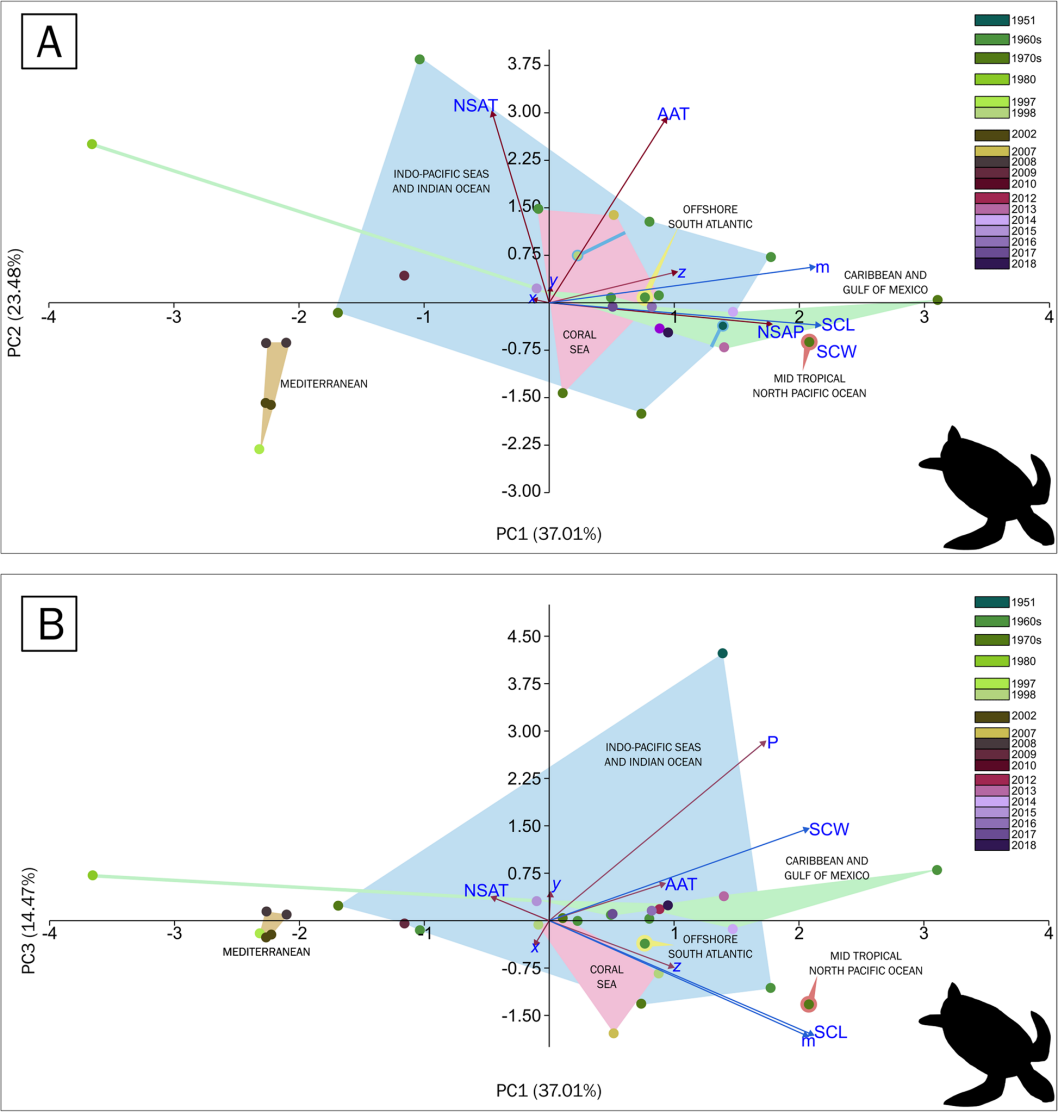
Principal component analysis biplots of the geoclimatic variables and hatchling sizes of *Chelonia mydas*. **A** Plotting PC1 against PC2 shows that the historical records can be separated into populations based on the following biogeographic marine realms: 8 from the Indo-Pacific seas and the Indian Ocean, with the widest spread over the plot; 10 from the Caribbean and Gulf of Mexico, mostly overlapping the Indo-Pacific seas and the Indian Ocean cluster; 4 from the Coral Sea; 5 from the Mediterranean; 1 from the Offshore South Atlantic; and 1 from the Mid-tropical North Pacific Ocean. The colour scale refers to the years where the samples were collected. **B** Plotting PC1 against PC3 showing the same clusters as described in (**A**)

In an initial exploration of the *C. mydas* dataset (Table 7), the z-axis was positively correlated with SCL (*p* = 0.04013, Table 8), whereas the AAT was positively correlated with mass (*p* = 0.0457, Table 8), and NSP was strongly positively correlated with SCW (*p* = 0.00026, Table 8). The first three components had eigenvalues greater than 1.0 and accounted for 74.96% of the variance, with PC1 mainly composed of hatchling size metrics (SCW-SCL-mass) (Fig. 6), PC2 of AAT and NSAT, and PC3 of NSP. When plotting PC1 vs. PC2 (Fig. 6A), SCL was strongly positively correlated with NSP. SCL and SCW are more strongly correlated with each other than with mass. The AAT and NSAT are likely not correlated with SCW or SCL but are slightly negatively correlated with mass. The z-axis is positively and strongly correlated with mass but less strongly correlated with SCL and SCW. Unlike in *C. caretta*, the clusters are not as clear. The Mediterranean populations are clustered together but are distinctly isolated from the rest of the populations. The Coral Sea populations and Indo-Pacific seas and Indian Ocean populations form overlapping clusters, whereas the Caribbean and Gulf of Mexico populations partially overlap with the former two. The offshore South Atlantic populations are nested within the main overlapping clusters, whereas the mid-tropical North Pacific Ocean is detached from all the clusters. Interestingly, the eggs were collected from the Caribbean and Gulf of Mexico populations in Tortuguero, Costa Rica, in 1980; the eggs were transported to the US and hatched under experimental conditions, and their placement too far from the cluster that corresponds to the Caribbean Sea and the Gulf of Mexico may reflect their growth without the precipitation regime in situ. When plotting PC1 vs. PC3 (Fig. 6B), the geographic clustering became clearer, with a Mediterranean cluster becoming increasingly detached. The z-axis is positively correlated with mass and SCL, but it is likely not related to SCW. The monthly average temperature during the nesting season was negatively correlated with mass and SCL, but the annual air temperature and precipitation were positively correlated with SCW.

**Fig. 7:**
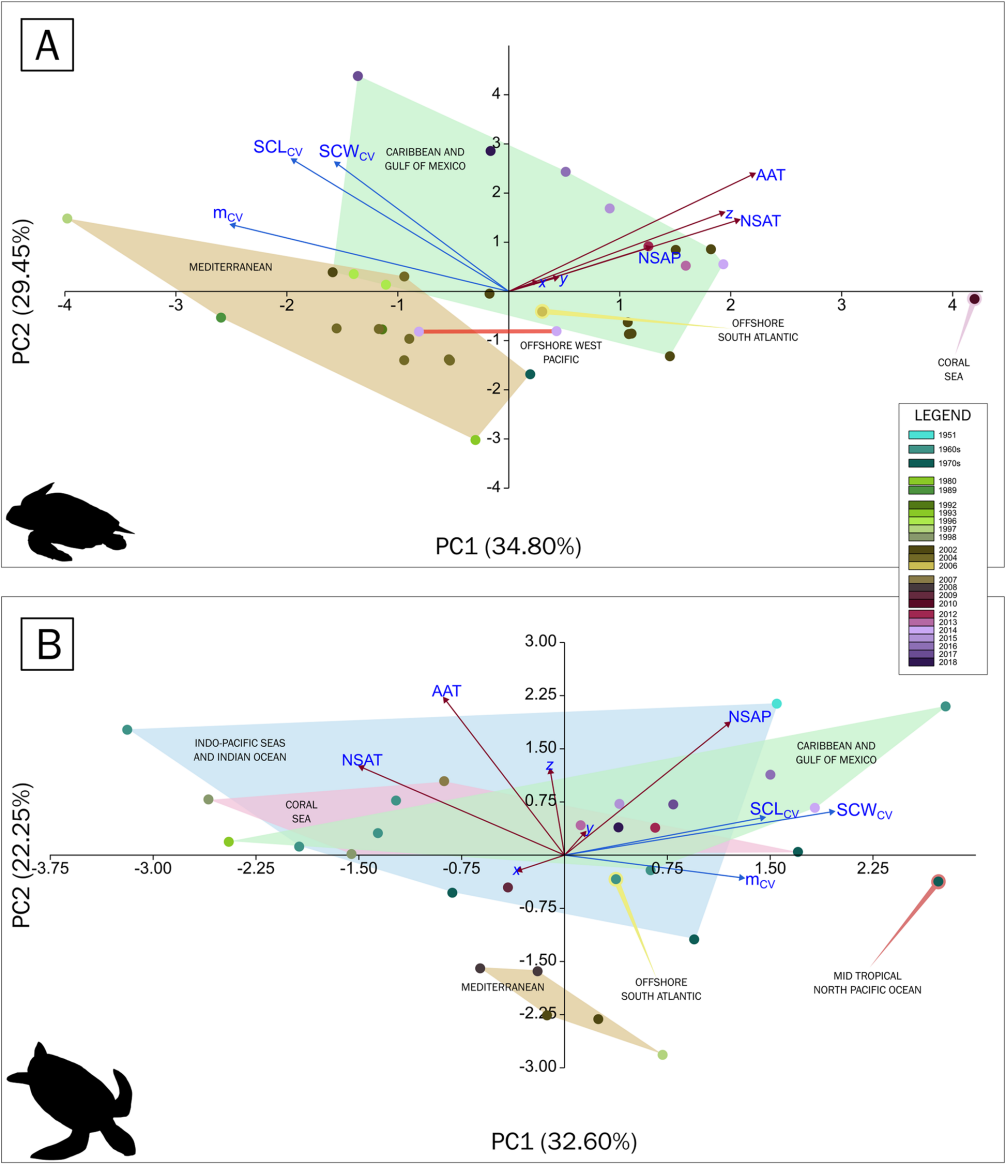
Biplots of the principal component analysis of the geoclimatic variables and the variation in the coefficient of variation of the hatchling size measurements (SCL, SCW and mass) of *Caretta caretta* (**A**) and *Chelonia mydas* (**B**)

In terms of coefficients of variation, the correlation matrix shows that the coefficient of variation of mass within the population is correlated with its distribution in the Eastern and Western Hemispheres (x-axis [*p* = 0.089] and y-axis [*p* = 0.054] negatively and positively correlated, respectively, albeit not significantly). The PCA from the *C. caretta* dataset produced two principal components that accounted for 64.25% of the variation (Fig. 7A), whereas the PCA from the *C. mydas* dataset produced two principal components that accounted for 54.85% of the variation (Fig. 7B).

In the case of *C. caretta*, PC1 contains mainly morphometric variables, whereas PC2 contains mainly geoclimatic variables. The coefficients of variation of mass, SCL and SCW are negatively correlated with the geoclimatic variables (Fig. 7A). The variation in mass is positively correlated with the variation in SCL and SCW. According to a global meta-analysis of *C. caretta*, the populations from the Mediterranean (Turkey [31, 38, 40, 43, 44], Northern Cyprus [41], Cyprus [38] and Greece [39]) are in a cluster opposite to the populations from the Atlantic coast of the USA [3, 42]. Although size (SCL-SCW-mass) appears to be related to temperature, i.e., the warmest temperatures occur closer to today, this is likely a geographic artefact where the Mediterranean populations are smaller than the American ones. The Mediterranean populations were sampled between 1978 and 2006, whereas the populations on the American Atlantic coast were sampled between 2002 and 2018 (Fig. 7A). This geographic pattern is clearly observed in the three samples taken from 2014, with the samples from the USA clustering togeher and those from the Japanese beaches straddling in the middle of the plot (Fig. 7A).

For *C. mydas*, the PCA of the geoclimatic variables against the coefficient of variation of hatchling size metrics does not show neatly separated groupings when considering the biogeographic realm [63]. The populations from the Indo-Pacific seas and Indian Ocean [2, 51–53], the Coral Sea, the Caribbean and Gulf of Mexico, and the offshore South Atlantic overlap with each other and have a similar spread over the PCA biplot when plotting PC1 vs. PC2 (Fig. 7B). The populations from the Mediterranean were the only ones separated from the rest, suggesting different variation trends when compared to the rest (Fig. 7B).

The influence of precipitation on the PCAs is greater for PC4, which has an eigenvalue less than 1.0. However, it explains the distribution of points along the PCA plot (Figs. 6 and 7). For instance, the two samples from 1973 were collected from two different islands in the Indian Ocean, namely, Europa [52] and Tromelin Island [53]. Europa Island is to the southwest of the coast of Madagascar. It receives less rainfall during the nesting season than does Tromelin Island to the northwest of the coast of Madagascar, which receives nearly three times as much monthly average cumulative rainfall. The European Plateau is to the left, where populations from Turkey [56] Northern Cyprus [41, 43], Yemen [49] and Australia [6, 55] are located, as well as the outlier removed from Tortuguero, Costa Rica, which, under laboratory conditions, was not subjected to any rainfall regime [54]. The in-situ study from Tortuguero, Costa Rica, clustered with the other Atlantic populations to the right, which were exposed to more rainfall [50]. Overall, this clustering shows that the geographic distribution plays a larger role in explaining the variance, i.e., reflecting distinct turtle populations with their own growth variability and their distinct response to the geoclimatic variables than the geoclimatic variables themselves on the species.

### Cabo Verde study on *C. caretta* hatchlings

**Fig. 8:**
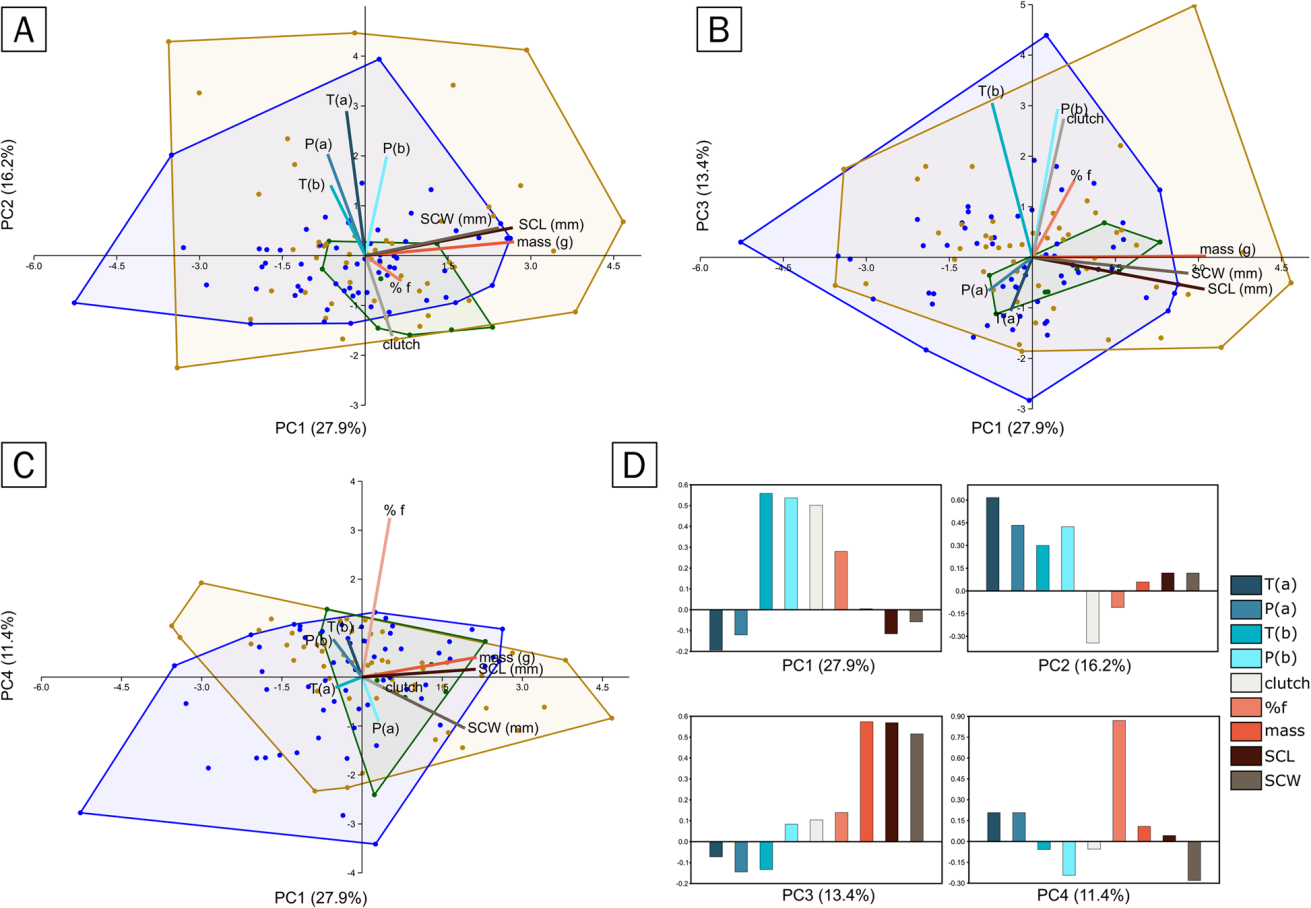
Biplots of the principal component analysis of the standardised geoclimatic variables and the standardised hatchling size of *C. caretta* collected from the beaches of Bõa Vista Island in Cabo Verde; missing data were automatically inputted via iterative permutation. The sampled nests are separated by colours representing the three different beaches from which they came (61 from Ponta Cosme, 49 from Ervatão, and 7 from Benguinho). **A** PC1 (27.9% of the variance) vs. PC2 (16.2% of the variance), **B** PC1 vs. PC3 (13.4% of the variance), **C** PC1 vs. PC4 (11.4% of the variance), **D** composition of the four PCs plotted in the previous panels, showing in cold colours the geoclimatic variables (temperature and precipitation at the mid-incubation period [T(a) and P(a)], temperature and precipitation two days before the mid-incubation period [T(b) and P(b)]) and in warm colours the hatchling size measurements (mass, SCL and SCW), in white the clutch size, and in salmon the estimated number of females

The eggs were laid between mid-July and mid-August, and they hatched between early September and mid-October 2020. August and September are often the hottest months, while July and August are the driest months. PCA (Fig. 8) revealed that three main components had eigenvalues greater than 1.0 and accounted for between 58.5% and 79.3% of the variance. According to the longitudinal data, the hatchling size and the triplet SCL-SCW mass are strongly positively correlated. According to the PCA plot, Porto Ervatão is more diverse in terms of hatchling size, whereas Ponta Benguinho has more similarly sized hatchlings. Most of the hatchlings from Ponta Cosme clustered near to each other.

When plotting PC1 (27.9% of the variance) vs. PC2 (16.2%), mass, SCL and SCW showed a weak positive correlation against geoclimatic variables. Of the two precipitation values, the precipitation two days before the mid-incubation period, P(b) in Fig. 8, was the driest, with a mean value of 0.27 mm, and showed a strong positive correlation with the hatchling size measurements, albeit weak. The other geoclimatic variables, precipitation in the middle of the incubation period, P(a) in Fig. 8, and temperature in the middle of the incubation period and two days before the mid-incubation period, T(a) and T(b) in Fig. 8, are likely not correlated with hatchling measurements. A stronger correlation between geoclimatic variables, namely, T(b) and P(b), is observed when plotting PC1 vs PC3 (13.4% of the variance). Furthermore, clutch size showed a strong positive correlation with P(b) in this biplot. Finally, PC4 (11.4% of the variance) is mostly composed of the estimated percentage of females in the nest, which suggests a weak correlation with any of the other variables. It is thus likely that the estimated number of females in the nests differed from the real number.

In *C. caretta*, hatchling size showed a strong negative correlation with precipitation (NSP) [corr(NSP,SCL) = -0.78, *p* = 0.0397; corr(NSP,SCW) = -0.91, *p* = 0.0047; corr(NSP,mass) = -0.86, *p* = 0.014] during the nesting season, whereas weight was strongly positively correlated with the average monthly air temperature during the nesting season (NSAT) [corr(NSAT,mass) = 0.84, *p* = 0.017]. The annual air temperature (AAT) is also strongly positively correlated with hatchling size, but this may be a confounding variable, as the average annual air temperature overall reflects the hydric and thermic conditions throughout the year [corr(AAT,SCL) = 0.85, *p* = 0.0142, corr(AAT,SCW) = 0.88, *p* = 0.0084, corr(AAT,mass) = 0.81, *p* = 0.0261]. In *C. mydas*, the environmental variables did not significantly correlate with hatchling size (Supplementary material). Other environmental factors may need to be investigated for *C. mydas*, such as the response of vegetation to drier and warmer conditions [64–66].

## Discussion

Sea turtle eggs remain unattended after nesting [67] and hatchlings have been shown to be influenced by air and sand temperatures in *C. mydas* [20, 23–25, 43] and *C. caretta* [19, 20, 26–28, 67–70]. For example, high incubation temperatures lead to an increase in the female-to-male ratio in *C. mydas* [71], and *C. caretta* [72], and incubation temperature can also influence the amount of yolk content, which in turn affects embryonic maturation, resulting in morphological differences in hatchlings in *C. caretta* [69].

Longitudinal data from Florida show that *C. caretta* and *C. mydas* respond differently to environmental conditions during their nesting seasons. Furthermore, the nesting periods of both species differ in terms of temperature and precipitation because *C. mydas* nest later, further from the high-water line, and closer to the vegetation [2, 64, 73], and they tend to miss much of the hottest, driest part of the summer.

The two distinct clusters of Mediterranean populations on the left of the PCA plot and the Atlantic and Indian Ocean populations on the right (Figs. 6 and 7) may correlate with the precipitation regime in both regions. The beaches in Cyprus and Turkey are generally dry during the nesting season of *C. caretta*, with months being completely dry [74]. Nevertheless, relative humidity, not included as a variable in this analysis, can reach very high values [74]. It has been proposed that precipitation affects the hatchling size of *C. caretta*. We posit that moisture can move into the egg and change the osmotic pressure inside, which facilitates the uptake of nutrients and the rate of organogenesis [18, 19, 75, 76]. During the second half of the incubation period, somatic growth follows the organogenesis, and a sped-up organogenesis could lead to prolonged somatic growth [18, 19, 75, 76]. More moisture means that the metabolism of the yolk is more efficient; when there is less moisture available, as in Mediterranean populations, fewer turtles hatch but with more yolk reserves due to slower metabolic rates, whereas with more moisture, as in the Atlantic Ocean populations, hatchlings are larger with fewer yolk reserves [16, 77, 78]. Thus, precipitation increases the variation in hatchling size.

The annual average air temperature was more strongly correlated with hatchling size than the average air temperature during the nesting season, but the precipitation during the nesting season had a greater ability to predict hatchling size according to our meta-analysis. Although precipitation is related to temperature, such that an increase in temperature may lead to an increase in precipitation, the rainfall levels of each region are also dependent on the topography, evaporation rates and vegetation cover [79]. The annual average temperature has a large predictive power because it is a better proxy of overall hydric conditions globally. The monthly average air temperature during the nesting season is less related to precipitation, given the other factors that influence the rainfall regime of a region. For instance, along the south-eastern coast of the USA, the evaporation rates of the Gulf of Mexico influence the amount of rain it receives each year [80], reflecting the rainfall drivers on the southwestern Florida coast, namely, sea breezes and land breezes and the daily heating of the Everglades affect the development of rainfall on the south-eastern Florida coast.

Variation in hatchling size was positively correlated with precipitation during the nesting season but negatively correlated with the annual and monthly average air temperatures. The extreme outliers in the PCA plots indicate that this may be due to the effect of sampling size. The population from Yemen collected in 1966 [49] displays a small variance compared to the population from the French Frigate Shoals collected in 1974; the former represents a sample of 20 individuals, and the latter represents a sample of 120 individuals [58]. The Mediterranean populations represent values collected from a sample of 175 individuals.

From the global meta-analyses described above, it is clear that the conditions at the nesting beach are strong indicators of hatchling size for both *C. caretta* and *C. mydas*. Precipitation during the nesting season is an indicator of the final hatchling size in the populations of both species. In the case of Florida populations, the relationship may decrease due to the climatic conditions of the region. During the winter–spring season, the global climatic patterns of ENSO in the Tropical Pacific, the North Atlantic and the Tropical Atlantic are sources of local precipitation for the Florida Peninsula [80], whereas during the summer–autumn, the local precipitation is sourced mostly from the Tropical Atlantic [80]. In the Mediterranean, on the other hand, the beaches of Cyprus and Turkey experience dry seasons with very little rain coming from the North Atlantic [81]. In drier environments, the effects of precipitation on *C. mydas* are observed when the dry season ends. In contrast, in Florida, the precipitation levels remain constant over time, given the sources of local precipitation, with droughts and heatwaves occurring in July and October in most years.

The observations of *C. caretta* in Cabo Verde indicate that the precipitation before the middle of the incubation period is a stronger predictor of hatchling size, which in our study was measured at the point where it is estimated to occur during the thermosensitive period.

A direct mechanism to explain this relationship is not readily obvious. One hypothesis could be that the precipitation affects the nesting temperature by lowering the temperature of the nests during the thermosensitive period, affecting the sex ratio, and the subtle sexual dimorphism in hatchlings as it is seen in other turtles, such as *Podocnemis expansa*, where it has been found that the male hatchlings have a more expanded central region of the carapace compared to females [82]. Precipitation may affect the sand temperature, cooling it down and increasing the number of males [43, 71, 72, 83]. However, the percentage of females was estimated from a gradient temperature, and it is not possible to confirm whether the larger individuals correspond to males, although this would be unlikely. Further study into the relationship between precipitation and hatchling size is required, as our experiment in Cabo Verde did not take into account topography. The clusters also indicate that the precipitation measured for Bõa Vista Island would be distributed differently among the three beaches, even when the weather stations are relatively close. Bõa Vista is the driest of all the islands of Cabo Verde, and stream flows would only occur with heavy rainfall: the streams are more likely to move towards the bay, Ervatão, than to the headland.

## Conclusions

From these three analyses, it is clear that the sea turtle populations of *C. caretta* and *C. mydas* are more susceptible to regional environmental variables than to global-scale environmental variables. Of these, precipitation is an important factor in the development of embryos. As hatchling body size is important as a defence mechanism to deter gape-limited predators during early ontogeny, differences in hatchling body size may change the type of predator that is attracted to hatchlings. Smaller sizes may lead to greater predation by otherwise gape-limited predators, yet larger sizes may attract larger predators on which predator satiation may not work.

As climate change alters rainfall regimes worldwide, with humid environments becoming wetter and arid environments becoming drier, the effects of precipitation on beaches may suggest that global strategies for the conservation of both *C. caretta* and *C. mydas* need to be revisited. Particularly, the concept of management conservation units has already shown that, at a molecular level, it requires the constant revaluation and inclusion of “key rookeries” [37, 84], emphasising the value of the local management and efforts at nesting sites. Analysis of local databases from different sea turtle rookeries is more important for understanding the dynamics of the nesting seasons for sea turtle populations, and these databases should become more widely available and easier to distribute, adding value to descriptive publications for local areas.

### Supplementary Information

Supplementary Material 1.

## Data Availability

Data is provided within the manuscript or supplementary information files.
